# Clinicopathological discrepancies in the diagnoses of childhood causes of death in the CHAMPS network: An analysis of antemortem diagnostic inaccuracies

**DOI:** 10.1136/bmjpo-2024-002654

**Published:** 2024-07-20

**Authors:** Haleluya Leulseged, Christine Bethencourt, Kitiezo Aggrey Igunza, Victor Akelo, Dickens Onyango, Richard Omore, Ikechukwu U Ogbuanu, Soter Ameh, Andrew Moseray, Dickens Kowuor, Ima-Abasi Bassey, Shams El Arifeen, Emily S Gurley, Mohammad Zahid Hossain, Afruna Rahman, Muntasir Alam, Nega Assefa, Lola Madrid, Addisu Alemu, Yasir Y Abdullahi, Karen L Kotloff, Samba O Sow, Milagritos D Tapia, Nana Kourouma, Seydou Sissoko, Quique Bassat, Rosauro Varo, Inacio Mandomando, Carla Carrilho, Natalia Rakislova, Fabiola Fernandes, Shabir Madhi, Ziyaad Dangor, Sana Mahtab, Martin Hale, Vicky Baillie, Jeanie du Toit, Zachary J Madewell, Dianna M Blau, Roosecelis B Martines, Portia C Mutevedzi, Robert F Breiman, Cynthia G Whitney, Chris A Rees, Child Health and Mortality Prevention Surveillance (CHAMPS) network, Fatima Solomon, Gillian Sorour, Hennie Lombaard, Jeannette Wadula, Karen Petersen, Nelesh P Govender, Peter J Swart, Sanjay G Lala, Sithembiso Velaphi, Richard Chawana, Yasmin Adam, Amy Wise, Ashleigh Fritz, Nellie Myburgh, Pedzisai Ndagurwa, Cleopas Hwinya, Sanwarul Bari, Shahana Parveen, Mohammed Kamal, ASM Nawshad Uddin Ahmed, Mahbubul Hoque, Saria Tasnim, Ferdousi Islam, Farida Ariuman, Mohammad Mosiur Rahman, Ferdousi Begum K Zaman, Mustafizur Rahman, Dilruba Ahmed, Meerjady Sabrina Flora, Tahmina Shirin, Mahbubur Rahman, Joseph Oundo, Alexander M Ibrahim, Fikremelekot Temesgen, Tadesse Gure, Melisachew Mulatu Yeshi, Mahlet Abayneh Gizaw, Stian MS Orlien, Solomon Ali, Peter Otieno, Peter Nyamthimba Onyango, Janet Agaya, Richard Oliech, Joyce Akinyi Were, Dickson Gethi, Sammy Khagayi, George Aol, Thomas Misore, Harun Owuor, Christopher Muga, Bernard Oluoch, Christine Ochola, Sharon M Tennant, Carol L Greene, Ashka Mehta, J Kristie Johnson, Brigitte Gaume, Adama Mamby Keita, Rima Koka, Karen D Fairchild, Diakaridia Kone, Sharon M Tennant, Carol L Greene, Ashka Mehta, Diakaridia Sidibe, Doh Sanogo, Uma U Onwuchekwa, Cheick Bougadari Traore, Jane Juma, Kounandji Diarra, Awa Traore, Tiéman Diarra, Kiranpreet Chawla, Tacilta Nhampossa, Zara Manhique, Sibone Mocumbi, Clara Menéndez, Khátia Munguambe, Ariel Nhacolo, Maria Maixenchs, Fatmata Bintu Tarawally, Martin Seppeh, Ronald Mash, Julius Ojulong, Babatunde Duduyemi, James Bunn, Alim Swaray-Deen, Joseph Bangura Amara Jambai, Margaret Mannah, Okokon Ita, Cornell Chukwuegbo, Sulaiman Sannoh, Princewill Nwajiobi, Erick Kaluma, Oluseyi Balogun, Carrie Jo Cain, Solomon Samura, Samuel Pratt, Francis Moses, Tom Sesay, James Squire, Joseph Kamanda Sesay, Osman Kaykay, Binyam Halu, Hailemariam Legesse, Francis Smart, Sartie Kenneh, Sartie Kenneh, Jana Ritter, Tais Wilson, Jonas Winchell, Jakob Witherbee, Navit T Salzberg, Jeffrey P Koplan, Margaret Basket, Ashutosh Wadhwa, Kyu Han Lee, Roosecelis Martines, Shamta Warang, Maureen Diaz, Jessica Waller, Shailesh Nair, Lucy Liu, Courtney Bursuc, Kristin LaHatte, Sarah Raymer, John Blevins, Solveig Argeseanu, Kurt Vyas, Manu Bhandari

**Affiliations:** 1 College of Health and Medical Sciences, Haramaya University, Harar, Ethiopia; 2 Hararghe Health Research, Haramaya University, Harar, Ethiopia; 3 London School of Hygiene & Tropical Medicine, London, UK; 4 Children's Hospital of Philadelphia Pediatrics Residency Program, Philadelphia, Pennsylvania, USA; 5 Center for Global Health Research, Kenya Medical Research Institute, Kisumu, Kenya; 6 Liverpool School of Tropical Medicine, Liverpool, UK; 7 Global Health Institute, Emory University, Atlanta, Georgia, USA; 8 Kisumu County Department of Health, Kisumu, Kenya; 9 Crown Agents, Freetown, Sierra Leone; 10 Hubert Department of Global Health, Rollins School of Public Health, Emory University, Atlanta, Georgia, USA; 11 Department of Community Medicine, University of Calabar, Calabar, Cross River, Nigeria; 12 Bernard Lown Scholars Program in Cardiovascular Health, Department of Global Health and Population, Harvard T.H. Chan School of Public Health, Boston, Massachusetts, USA; 13 International Centre for Diarrhoeal Disease Research Bangladesh, Dhaka, Bangladesh; 14 Department of Epidemiology, Johns Hopkins Bloomberg School of Public Health, Baltimore, Maryland, USA; 15 Department of Pediatrics, University of Maryland School of Medicine, Baltimore, Maryland, USA; 16 Centre pour le Développement des Vaccins-Mali, Bamako, Mali; 17 Centro de Investigação em Saúde de Manhiça [CISM], Maputo, Mozambique; 18 ISGlobal - Hospital Clínic, Universitat de Barcelona, Barcelona, Spain; 19 ICREA, Pg. Lluís Companys 23, 08010, Barcelona, Spain; 20 Pediatrics Department, Hospital Sant Joan de Déu, Universitat de Barcelona, Esplugues, Barcelona, Spain; 21 CIBER de Epidemiología y Salud Pública, Instituto de Salud Carlos III, Madrid, Spain; 22 Department of Pathology, Maputo Central Hospital, Maputo, Mozambique; 23 Department of Pathology, Faculty of Medicine, Eduardo Mondlane University, Maputo, Mozambique; 24 South African Medical Research Council Vaccines and Infectious Diseases Analytics Research Unit, University of the Witwatersrand, Johannesburg, South Africa; 25 Department of Anatomical Pathology, University of the Witwatersrand Johannesburg, Johannesburg, South Africa; 26 Center for Global Health, Centers for Disease Control and Prevention, Atlanta, Georgia, USA; 27 Infectious Diseases Pathology Branch, NCEZID, DHCPP, Centers for Disease Control and Prevention, Atlanta, Georgia, USA; 28 Infectious Diseases and Oncology Research Institute, University of the Witwatersrand, Johannesburg, South Africa; 29 Pediatric Emergency Medicine, Emory University School of Medicine, Atlanta, Georgia, USA

**Keywords:** Child Health, Mortality

## Abstract

**Introduction:**

Determining aetiology of severe illness can be difficult, especially in settings with limited diagnostic resources, yet critical for providing life-saving care. Our objective was to describe the accuracy of antemortem clinical diagnoses in young children in high-mortality settings, compared with results of specific postmortem diagnoses obtained from Child Health and Mortality Prevention Surveillance (CHAMPS).

**Methods:**

We analysed data collected during 2016–2022 from seven sites in Africa and South Asia. We compared antemortem clinical diagnoses from clinical records to a reference standard of postmortem diagnoses determined by expert panels at each site who reviewed the results of histopathological and microbiological testing of tissue, blood, and cerebrospinal fluid. We calculated test characteristics and 95% CIs of antemortem clinical diagnostic accuracy for the 10 most common causes of death. We classified diagnostic discrepancies as major and minor, per Goldman criteria later modified by Battle.

**Results:**

CHAMPS enrolled 1454 deceased young children aged 1–59 months during the study period; 881 had available clinical records and were analysed. The median age at death was 11 months (IQR 4–21 months) and 47.3% (n=417) were female. We identified a clinicopathological discrepancy in 39.5% (n=348) of deaths; 82.3% of diagnostic errors were major. The sensitivity of clinician antemortem diagnosis ranged from 26% (95% CI 14.6% to 40.3%) for non-infectious respiratory diseases (eg, aspiration pneumonia, interstitial lung disease, etc) to 82.2% (95% CI 72.7% to 89.5%) for diarrhoeal diseases. Antemortem clinical diagnostic specificity ranged from 75.2% (95% CI 72.1% to 78.2%) for diarrhoeal diseases to 99.0% (95% CI 98.1% to 99.6%) for HIV.

**Conclusions:**

Antemortem clinical diagnostic errors were common for young children who died in areas with high childhood mortality rates. To further reduce childhood mortality in resource-limited settings, there is an urgent need to improve antemortem diagnostic capability through advances in the availability of diagnostic testing and clinical skills.

**WHAT IS ALREADY KNOWN ON THIS TOPIC**

- A significant proportion (58%–77%) of deaths among young children in sub-Saharan Africa and South Asia are preventable with timely access to high-quality clinical care, which includes accurate diagnoses.

**WHAT THIS STUDY ADDS**

- Using data from 881 deaths among infants and children aged 1–59 months from the 10 most common causes determined through extensive postmortem examinations in 7 countries, we documented antemortem diagnostic errors in 39.5% of cases, with 82.3% of those diagnostic errors being considered major.
- To further reduce childhood mortality in resource-limited settings, there is an urgent need to improve antemortem diagnostic capability.

**HOW THIS STUDY MIGHT AFFECT RESEARCH, PRACTICE OR POLICY**

- Enhancing antemortem diagnostic capacity is a crucial step towards directing appropriate clinical care to children in regions with high childhood mortality rates in sub-Saharan Africa and South Asia.
- The comparison of common antemortem clinical diagnostic errors compared with postmortem determined causes of death identifies the disease processes in most need of improved diagnostic approaches.

## Introduction

 Despite more than a 50% reduction in childhood mortality rates since the 1990s, an estimated 5.2 million deaths occurred among children aged <5 years in 2019.^1^ Over 80% of deaths among children aged <5 years occur in sub-Saharan Africa and South Asia.^2^ Disparate rates in childhood mortality are attributable, in part, to high rates of childhood malnutrition, infections, poverty, and poor access to the health system, which are common in sub-Saharan Africa and South Asia.^3^

In addition to these factors that contribute to high rates of childhood mortality in these regions, prior studies suggest that as much as 58%–77% of childhood deaths in resource-limited settings could have potentially been averted with more timely access to high-quality clinical care.^4 5^ Limited access to laboratory and radiographic diagnostic tests, resulting in inaccurate diagnoses and subsequent inappropriate patient treatment contribute to high rates of childhood mortality in sub-Saharan Africa and South Asia.^6^,^8^ In a previous study, when antemortem and postmortem diagnoses aligned, clinician adherence to clinical care recommendations was higher.^9^ Thus, making correct diagnoses is key to improving clinical care in resource-limited settings with high rates of childhood mortality.

A comparison of antemortem clinical diagnoses to postmortem-determined causes of death may help identify the most relevant diagnostic improvements to potentially reduce deaths among children aged <5 years. Previous studies suggest that rates of discrepancies in antemortem clinical diagnoses and postmortem diagnoses range from 20% of childhood deaths in the USA and Chile to 38% of maternal deaths in Mozambique and 40% of deceased adults in South America, using complete diagnostic autopsy as the reference standard.^10^,^13^ However, studies on the accuracy of antemortem diagnoses among young children are lacking in regions with high rates of childhood mortality in sub-Saharan Africa and South Asia.

Our objective was to evaluate antemortem clinical diagnostic accuracy compared with causes of death determined by postmortem investigation among infants and children aged 1–59 months who died in seven sites in sub-Saharan Africa and South Asia participating in Child Health and Mortality Prevention Surveillance (CHAMPS). Moreover, we aimed to describe factors associated with major (ie, an accurate diagnosis could have impacted survival) antemortem diagnostic errors compared with postmortem causes of death. Such an understanding is a necessary step towards health systems’ strengthening and allocating the most crucial diagnostic resources to correctly diagnose children, improve clinical outcomes, and potentially reduce childhood mortality.

## Methods

### Study design

We conducted a descriptive study and analysed data from seven sites in sub-Saharan Africa and Bangladesh participating in the CHAMPS Network. CHAMPS is conducting prospective mortality surveillance in healthcare centres and communities in areas of high mortality among children aged <5 years (ie, >50 per 1000 live births).^14 15^

### Patient and public involvement

The development of the research question was informed by the large burden child mortality worldwide. Patients were not involved in the design, recruitment or conduct of the study. Results of this study will be made publicly available through publication where study participants may access them. Patients were not advisers in this study.

### Study setting

The CHAMPS Network was established in 2015 and includes sites in Baliakandi and Faridpur, Bangladesh; Kersa, Haramaya and Harar, Ethiopia; Kisumu and Siaya, Kenya; Bamako, Mali; Manhiça and Quelimane, Mozambique; Makeni and Bo, Sierra Leone; and Soweto, South Africa.^16^ These regions were selected because they have high rates of mortality among children aged <5 years. CHAMPS employs postmortem pathological and microbiological evaluations to ascertain causes of child deaths, with the aim of preventing deaths by informing policies and programmes.^17^

### Population and inclusion and exclusion criteria

Our study population included all infants and children aged 1–59 months who died and were enrolled in CHAMPS from December 2016 to February 2022 and had documented antemortem clinical diagnoses. CHAMPS enrols stillbirths and deaths among neonates, infants and children that occur in health facilities or in the community. Here, we focused our analyses on deceased infants and children (1) with identifiable antemortem clinical diagnoses within their clinical records, (2) with consenting caregivers, (3) who underwent minimally invasive tissue sampling (MITS), and had adequate samples to determine causes of death and (4) whose results were reviewed by expert panels to determine postmortem pathology-based diagnoses. We excluded cases without clinical records and those who died before presentation to a health facility because antemortem clinical diagnoses could not be determined for these deaths.

### Study procedures

Surveillance for stillbirths and child deaths occurring in healthcare facilities and the community is performed by CHAMPS staff at each site. CHAMPS enrols cases within 24 hours of death for the MITS procedure. Within this time frame, caregivers of deceased neonates, infants and children are informed about the CHAMPS data collection process and approached for potential enrolment in the study.^15 18^ CHAMPS procedures include the collection of demographic information, clinical record review, verbal autopsy, and extensive postmortem tissue sampling and testing. Briefly, MITS consists of collection of tissue samples from brain, lungs, liver, and heart using biopsy needles, in addition to samples of blood and cerebrospinal fluid using syringes. Moreover, nasopharyngeal and rectal swabs are collected. MITS serves as a proxy for complete diagnostic autopsy and has demonstrated high concordance, for all age groups, with complete diagnostic autopsy.^19^,^21^ All collected samples undergo histopathological and microbiological analyses including H&E staining, targeted histochemistry, and immunohistochemistry dictated by the morphological findings and molecular pathogen identification through extensive PCR testing for 126 different pathogens (TaqMan array; ThermoFisher Scientific, Waltham, Massachusetts, USA).^17 22^

Tissues and body fluids are sent for culture. Blood samples also undergo HIV PCR testing, malaria thick and thin smears, and TaqMan array PCR testing (including the screening of 126 pathogens) and microbial culture.^22^ Nasopharyngeal, oropharyngeal, and rectal swabs are sent for TaqMan array PCR testing. Samples are reviewed by anatomical pathologists who have received specific training for the CHAMPS study at each site, as well as by a central pathology lab at the US Centers for Disease Control and Prevention.^23^

To comprehensively capture clinicians’ diagnostic considerations and to determine antemortem clinical diagnoses, CHAMPS staff extracted all diagnoses recorded during the last clinical encounter before the infant’s or child’s death. These data were abstracted from all available admission forms, progress notes, laboratory results, and radiographic test results.

All clinical, verbal autopsy, histopathological, and microbiological results were subsequently reviewed through a standardised process by CHAMPS Determination of Cause of Death (DeCoDe) panels, which are composed of experts across various fields, including paediatricians, pathologists, microbiologists, medical epidemiologists, local public health officials, and representatives of local ministries of health.^17^ They use antemortem clinical data, which includes clinicians’ documentation of clinical history, progress notes, laboratory and radiographic imaging results, postmortem MITS pathological results, microbiological results, and verbal autopsy data to determine the underlying cause of death, as well as the comorbid and immediate conditions contributing to death in all enrolled cases. The clinical diagnoses considered as antemortem diagnoses included all diagnoses documented from the time of the child’s clinical encounter to the time of the child’s death, which included diagnoses at admission (including known chronic diseases such as HIV), interim diagnoses and final diagnoses. To comprehensively capture all documented causes for each case of death, we analysed all levels of diagnoses along the causal chain of mortality. The causal chain of mortality refers to all causes of death documented for each case, including the underlying cause of death, immediate cause of death that directly preceded terminal events, and comorbid conditions contributing to death. Determined causes of death are coded in agreement with the International Classification of Diseases, 10th Revision and the WHO death certificate.^17^

### Variables

Each documented antemortem clinical diagnosis was compared with causes of death as determined by DeCoDe panels, which served as the reference standard. Overall, the 10 most common causes of death as determined by DeCoDe panels based on the aggregated CHAMPS data were (1) lower respiratory infections, (2) sepsis, (3) malnutrition, (4) malaria, (5) diarrhoeal diseases, (6) HIV, (7) congenital birth defects, (8) anaemia, (9) other respiratory disease (ie, non-infectious syndromes including aspiration pneumonia, interstitial lung disease and pulmonary haemorrhage), and (10) meningitis/encephalitis.^24^

Each documented antemortem clinical diagnosis was paired with each postmortem cause of death for every case. For cases with multiple postmortem causes of death, all postmortem causes of death were included (ie, immediate, underlying or comorbid). We classified each potential antemortem and postmortem diagnostic pairing that were discrepant as major (class I or class II) or minor (class III or class IV) diagnostic errors, following the criteria originally developed by Goldman and later modified by Battle.^25 26^ Two authors with clinical training in paediatrics (CB and CAR) independently reviewed each diagnostic pairing to classify them according to the established system. Any disagreements between these two reviewers were discussed until consensus was reached for each diagnostic pairing. Class I errors included errors in which ‘knowledge of the correct diagnosis before death would have impacted clinical management decisions that could have resulted in either cure or prolonged survival’ (eg, sepsis treated as malnutrition).^25 26^ Class II diagnostic errors were those in which the impact on survival was equivocal despite the misdiagnosis for primary diagnoses, such as cases in which there was ambiguity on whether the correct diagnosis would have changed outcomes^26^ (eg, lower respiratory tract infections treated as sepsis). Class III included missed diagnoses with symptoms that should have been treated because they were either related to the terminal disease but not directly responsible for death or could have eventually impacted prognosis^25^ (eg, congenital birth defects treated as anaemia). Class IV diagnostic discrepancies included undiagnosed diseases that carried either genetic or epidemiological importance and may have eventually impacted prognosis^26^ (eg, sickle cell disease treated as anaemia alone). Correctly diagnosed patients were categorised as class V. Deaths that were non-classifiable due to unclear antemortem clinical diagnoses or insufficient postmortem information were categorised as class VI.

### Statistical analyses

Sensitivity, specificity and area under the curve (AUC) of each antemortem clinical diagnosis were calculated compared with each postmortem cause of death. We calculated the positive predictive value, negative predictive value, positive likelihood ratio, negative likelihood ratio, proportion correctly diagnosed, and apparent and true prevalence for antemortem diagnoses for each cause of death. We used Cohen’s Kappa to determine agreement between antemortem diagnoses and postmortem-determined causes of death.^27^ For deaths with multiple antemortem clinical diagnoses or multiple postmortem causes of death, each antemortem diagnosis was compared with each postmortem cause of death and considered a match if the antemortem clinical diagnosis was in any location in the causal chain determined by the expert panel at each site.

To identify the diseases in which antemortem clinical diagnoses had the most optimal sensitivity and specificity, we calculated the Youden’s index,^28^ which allows for the quantification of the overall diagnostic performance. We calculated the number needed to diagnose, which is the number of patients that need to be diagnosed antemortem to identify one correctly diagnosed patient. We calculated 95% CIs for all measures. We used mixed-effect univariable and multivariable logistic regression to identify factors associated with major diagnostic errors, including age, sex, time from hospital admission to death, the number of postmortem diagnoses, and site as candidate variables. The selection of these candidate variables for the inclusion in the model was predicated on their potential influence on diagnostic errors. Age, for instance, might influence disease presentation in children while the time from admission to death could impact disease progression and recognition of signs and symptoms. The number of postmortem diagnoses and site were included due to potential variations in healthcare infrastructure, diagnostic capabilities and disease prevalence across different regions. As diagnostic capacity and patient complexity may vary across CHAMPS sites, we introduced an interaction term between site and number of causes of death. We also conducted sensitivity analyses to determine the test characteristics of antemortem clinician diagnosis to postmortem determined causes of death by site, sex, age (ie, 1–11 months and 12–59 months) and duration of hospital admission (ie, <24 hours and ≥24 hours). All statistical analyses were performed by using R software, V.4.3.1 (R Foundation for Statistical Computing).

## Results

CHAMPS enrolled 1454 deaths in infants and children aged 1–59 months from December 2016 to February 2022. Of these, 498 did not have antemortem clinical records available and 75 died prior to presentation at a health facility, leaving 881 (60.5%) deaths that were included in the analyses (online supplemental figure 1). Included cases did not differ from excluded cases by age or sex but differed by site of enrolment (online supplemental table 1). The median age at the time of death was 11 months (IQR 4–21 months) and 47.3% (n=417) were female. The majority (74.6%, n=657) died in hospital and the other 224 (25.4%) died at home following clinical encounters. Cases were distributed across the seven sites as follows: Kenya (25.9%, n=228), Sierra Leone (24.4%, n=215), South Africa (18.6%, n=164), Mali (15.3%, n=135), Mozambique (10.0%, n=88), Bangladesh (3.3%, n=29) and Ethiopia (2.5%, n=22) (table 1).

**Table 1:** Description of deceased infants and children included in the analyses and causes of death identified by Child Health and Mortality Prevention Surveillance determination of cause of death process (N=881) who died from 2016 to 2022

	n (%)
Age in months (median, IQR)*	11 (4–21)
Female	417 (47.3)
Site	
Bangladesh	29 (3.3)
Ethiopia	22 (2.5)
Kenya	228 (25.9)
Mali	135 (15.3)
Mozambique	88 (10.0)
Sierra Leone	215 (24.4)
South Africa	164 (18.6)
Most common antemortem clinician diagnoses	
Malaria	280 (31.8)
Diarrhoeal disease	270 (30.7)
Lower respiratory tract infections	269 (30.5)
Malnutrition	199 (22.6)
Anaemia	170 (19.3)
Sepsis	157 (17.8)
Severe dehydration	91 (8.5)
Congenital birth defects	81 (9.2)
Meningitis	68 (7.7)
Seizure	58 (6.6)
Most common postmortem determined causes of death anywhere in the causal chain of death	
Lower respiratory tract infections	274 (31.1)
Sepsis	258 (29.3)
Malnutrition	167 (19.0)
Malaria	142 (16.1)
Diarrhoeal diseases	90 (10.2)
HIV	68 (7.7)
Congenital birth defects	63 (7.2)
Anemias	93 (10.6)
Other respiratory disease†	50 (5.7)
Meningitis/encephalitis	49 (5.6)

* Two cases had missing age but were categorizedcategorised as infants or children at the time of death.

† Non-infectious respiratory conditions such aspiration pneumonia, interstitial lung disease, and pulmonary hemorrhagehaemorrhage.

### Antemortem clinical diagnoses

The most common antemortem diagnoses among the included cases were malaria (31.8%, n=280), diarrhoeal disease (30.7%, n=270), lower respiratory tract infections (30.5%, n=269) and malnutrition (22.6%, n=199) (table 1).

### Postmortem determined causes of death

Overall, the most common causes of death anywhere in the causal chain of death were lower respiratory tract infections (31.1%, n=274), sepsis (29.3%, n=258), and malnutrition (19.0%, n=167; table 1). The most common underlying causes of death determined through postmortem examinations were malnutrition (13.5%, n=119), malaria (8.7%, n=77), and HIV (7.7%, n=68) (online supplemental table 2). The most common immediate causes of death were sepsis (21.5%, n=189), lower respiratory tract infections (14.2%, n=125), and malaria (5.6%, n=49). Lower respiratory tract infections (12.5%, n=110), sepsis (6.6%, n=58), and anaemias (7.2%, n=63) were the most common comorbid causes of death. When there was only one cause of death determined postmortem, the most common conditions were malaria (n=43), lower respiratory infections (n=22), and diarrhoeal diseases (n=17).

### Test characteristics of antemortem clinical diagnoses

A total of 635 diagnostic pairs between antemortem clinician diagnosis and postmortem causes of death were concordant. There were 64 diagnostic pairs between antemortem clinician diagnosis and postmortem causes of death that were unclassifiable. Malaria and diarrhoeal diseases were most often correctly diagnosed in the antemortem period (online supplemental table 3). Anaemias, meningitis/encephalitis, and respiratory diseases other than lower respiratory tract infections (ie, aspiration pneumonia, interstitial lung disease and pulmonary haemorrhage) were most infrequently diagnosed among the top 10 causes of death.

**Figure 1:**
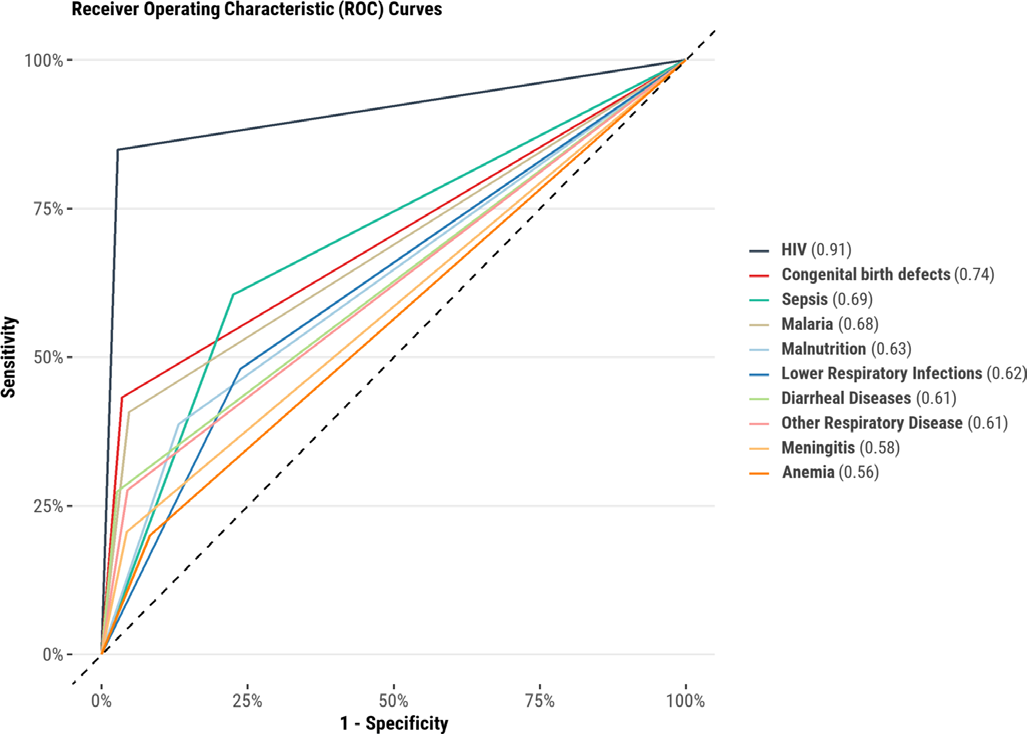
Receiver operating characteristic curves for antemortem clinical diagnoses compared with postmortem causes of death among infants and children aged 1–59 months who died in one of seven sites in the Child Health and Mortality Prevention Surveillance network (data collected during 2016–2022). The area under the curve provides an aggregate measure of diagnostic precision, with 1.0 representing perfect discriminatory ability and 0.5 suggesting worthless discrimination.

The sensitivity of antemortem clinical diagnosis ranged from 26.0% (95% CI 14.6% to 40.3%) for the category of other respiratory diseases to 82.2% (95% CI 72.7% to 89.5%) for diarrhoeal diseases (table 2). The specificity of antemortem clinical diagnosis ranged from 75.2% (95% CI 72.1% to 78.2%) for diarrhoeal diseases to 99.0% (95% CI 98.1% to 99.6%) for HIV (table 2). The AUC for antemortem clinical diagnoses compared with postmortem determined cause of death ranged from 0.56 for anaemias to 0.91 for HIV (figure 1).

**Table 2:** Comparison of clinician antemortem diagnosis and postmortem causes of death for N=881 deaths among infants and children aged 1–59 months enrolled in CHAMPS sites from 2016 to 2022

Cause of death determined postmortem by CHAMPS	Test characteristics of clinician antemortem diagnosis compared with postmortem determined cause of death								
	True positives, n (%)	True negatives, n (%)	False positives, n (%)	False negatives, n (%)	Sensitivity % (95% CI)	Specificity % (95% CI)	Positive predictive value % (95% CI)	Negative predictive value % (95% CI)	Cohen’s Kappa (95% CI)
Lower respiratory tract infection	130 (14.8)	466 (52.9)	141 (16.0)	144 (16.3)	47.4 (41.4 to 53.5)	76.8 (73.2 to 80.1)	48.0 (41.9 to 54.1)	76.4 (72.8 to 79.7)	0.24 (0.17 to 0.31)
Sepsis	96 (10.9)	560 (63.6)	63 (7.2)	162 (18.4)	37.2 (31.3 to 43.4)	89.9 (87.2 to 92.1)	60.4 (52.3 to 68.0)	77.6 (74.3 to 80.6)	0.30 (0.23 to 0.37)
Malnutrition	77 (8.7)	592 (67.2)	122 (13.8)	90 (10.2)	46.1 (38.4 to 54.0)	82.9 (79.9 to 85.6)	38.7 (31.9 to 45.8)	86.8 (84.0 to 89.3)	0.27 (0.20 to 0.34)
Malaria	114 (12.9)	572 (64.9)	167 (19.0)	28 (3.2)	80.3 (72.8 to 86.5)	77.4 (74.2 to 80.4)	40.6 (34.8 to 46.6)	95.3 (93.3 to 96.9)	0.42 (0.35 to 0.48)
Diarrhoeal diseases	74 (8.4)	595 (67.5)	196 (22.2)	16 (1.8)	82.2 (72.7 to 89.5)	75.2 (72.1 to 78.2)	27.4 (22.2 to 33.1)	97.4 (95.8 to 98.5)	0.30 (0.24 to 0.37)
HIV	45 (5.1)	805 (91.4)	8 (0.9)	23 (2.6)	66.2 (53.7 to 77.2)	99.0 (98.1 to 99.6)	84.9 (72.4 to 93.3)	97.2 (95.9 to 98.2)	0.73 (0.63 to 0.82)
Congenital birth defects	35 (4.0)	772 (87.6)	46 (5.2)	28 (3.2)	55.6 (42.5 to 68.1)	94.4 (92.6 to 95.9)	43.2 (32.2 to 54.7)	96.5 (95.0 to 97.7)	0.44 (0.34 to 0.55)
Anaemias	34 (3.9)	652 (74.0)	136 (15.4)	59 (6.7)	36.6 (26.8 to 47.2)	82.7 (79.9 to 85.3)	20.0 (14.3 to 26.8)	91.7 (89.4 to 93.6)	0.14 (0.07 to 0.22)
Other respiratory diseases*	13 (1.5)	797 (90.5)	34 (3.9)	37 (4.2)	26.0 (14.6 to 40.3)	95.9 (94.3 to 97.2)	27.7 (15.6 to 42.6)	95.6 (93.9 to 96.9)	0.23 (0.11 to 0.35)
Meningitis/encephalitis	14 (1.6)	778 (88.3)	54 (6.1)	35 (4.0)	28.6 (16.6 to 43.3)	93.5 (91.6 to 95.1)	20.6 (11.7 to 32.1)	95.7 (94.1 to 97.0)	0.19 (0.08 to 0.29)

* Includes aspiration pneumonia, interstitial lung disease,, and pulmonary hemorrhagehaemorrhage.

CHAMPSChild Health and Mortality Prevention Surveillance

The positive likelihood ratio of clinician antemortem diagnoses ranged from 2.12 (95% CI 1.56 to 2.88) for anaemias to 67.25 (95% CI 33.06 to 136.81) for HIV (online supplemental table 4). Negative likelihood ratios of clinician antemortem diagnoses ranged from 0.25 (95% CI 0.18 to 0.36) for malaria to 0.77 (95% CI 0.65 to 0.91) for other respiratory diseases. The overall diagnostic accuracy of clinician antemortem diagnoses was highest for HIV (Youden’s index 0.65, 95% CI 0.52 to 0.77) and lowest for anaemias (Youden’s index 0.19, 95% CI 0.07 to 0.33). There were 464 cases in our analyses that had >1 postmortem-determined cause of death. Of these, the majority (83.6%, n=388) did not have all postmortem causes of death diagnosed prior to the child’s death.

### Major and minor diagnostic discrepancies

A discrepancy between antemortem clinical diagnosis and postmortem determined causes of death was identified in 39.5% (n=348/881) cases. A total of 555 diagnostic errors were identified among 1254 diagnostic comparisons between antemortem clinical diagnoses and postmortem causes of death (table 3). Among the 555 diagnostic errors, 457 (82.3%) were classified as major and 98 (17.7%) as minor. Major antemortem diagnostic errors were most common among infants and children who died from meningitis/encephalitis (63.3%, n=31) and sepsis (54.2%, n=140), and least common among diagnostic pairs in cases of congenital birth defects (3.2%, n=2) and malaria (13.4%, n=19) (table 3). Minor diagnostic errors were most common among cases that died from anaemias (43.0%, n=40) and congenital birth defects (31.7%, n=20).

**Table 3:** Distribution of antemortem clinical diagnostic errors by class for the top 10 causes of death among infants and children aged 1–59 months enrolled in CHAMPS sites from 2016 to 2022

Postmortem CHAMPS determined cause of death	Major errors		Minor errors		No error	Could not determine	Total, n
	Class I,*n (%)	Class II,†n (%)	Class III,‡n (%)	Class IV,§n (%)	Class V,¶n (%)	Class VI,**n (%)	
Lower respiratory tract infection	86 (31.4)	33 (12.0)	11 (4.0)	2 (0.7)	128 (46.7)	14 (5.1)	274
Sepsis	127 (49.2)	13 (5.0)	6 (2.3)	4 (1.6)	96 (37.2)	12 (4.7)	258
Malnutrition	76 (45.5)	0 (0)	0 (0)	0 (0)	78 (46.7)	13 (7.8)	167
Malaria	19 (13.4)	0 (0)	0 (0)	1 (0.7)	113 (79.6)	9 (6.3)	142
Anaemias	15 (16.1)	0 (0)	0 (0)	40 (43.0)	35 (37.6)	3 (3.2)	93
Diarrhoeal diseases	15 (16.7)	0 (0)	0 (0)	0 (0)	74 (82.2)	1 (1.1)	90
HIV	17 (25.0)	0 (0)	0 (0)	1 (1.5)	45 (66.2)	5 (7.4)	68
Congenital birth defects	2 (3.2)	0 (0)	0 (0)	20 (31.7)	39 (61.9)	2 (3.2)	63
Other respiratory diseases††	22 (44.0)	1 (2.0)	9 (18.0)	2 (4.0)	13 (26.0)	3 (6.0)	50
Meningitis/encephalitis	22 (44.9)	9 (18.4)	1 (2.0)	1 (2.0)	14 (28.6)	2 (4.1)	49

* Errors that may have impacted survival if properly diagnosed/treated.

† Errors in which impact on survival was equivocal despite the misdiagnosis for primary diagnoses.

‡ Missed diagnoses not directly responsible for death.

§ Missed diagnoses with genetic/epidemiological importance.

¶ Antemortem and postmortem diagnoses aligned.

** Deaths that were non-classifiable due to unclear antemortem clinical diagnoses or insufficient postmortem information.

†† Includes aspiration pneumonia, interstitial lung disease, and pulmonary hemorrhagehaemorrhage.

CHAMPSChild Health and Mortality Prevention Surveillance

In multivariable analyses, infants and children who had 2–3 postmortem causes of death (adjusted OR (aOR) 33.9, 95% CI 17.4 to 73.2) and >3 postmortem causes of death (aOR 90.5, 95% CI 42.7 to 210.3) were more likely to have a major antemortem diagnostic error than those who had only one postmortem cause of death (table 4). The interaction term between site and the number of causes of death did not reach statistical significance (p=0.428), which suggests that number of causes of death exerted an effect independent of site. Age, sex, duration of hospital admission and site were not associated with the presence of a major diagnostic error.

**Table 4:** Crude and adjusted analyses of factors associated with major antemortem diagnostic errors (N=636*)

	Minor or no diagnostic error, n (%)N=381	Major diagnostic error, n (%)N=255	OR (95% CI)	P value	Adjusted OR (95% CI)	P value
Age at the time of death				0.807		0.985
1–11 months	198 (52.0)	130 (51.0)	*Referent*		--	
12–59 months	183 (48.0)	125 (49.0)	1.04 (0.76 to 1.43)		1.00 (0.65 to 1.53)	
Sex				0.785		0.365
Male	199 (52.2)	136 (53.3)	1.05 (0.76 to 1.44)		1.21 (0.80 to 1.83)	
Female	182 (47.8)	119 (46.7)	*Referent*		--	
Time from admission to death				0.163		0.378
<24 hours	163 (42.8)	95 (37.3)	*Referent*		--	
≥24 hours	218 (57.2)	160 (62.7)	1.26 (0.91 to 1.75)		0.81 (0.51 to 1.28)	
Number of postmortem diagnoses				<0.001		<0.001
1	240 (63.0)	10 (3.9)	*Referent*		*Referent*	
2–3	108 (28.3)	136 (53.3)	30.22 (16.03 to 63.45)		33.90 (17.42 to 73.18)	
>3	33 (8.7)	109 (42.7)	79.27 (39.38 to 175.96)		90.48 (42.73 to 210.25)	
Site				<0.001		0.477
Bangladesh†	5 (1.3)	1 (0.4)	--		--	
Ethiopia†	4 (1.0)	5 (2.0)	--		--	
Kenya	80 (21.0)	51 (20.0)	0.54 (0.34 to 0.87)		1.41 (0.78 to 2.58)	
Mali	60 (15.7)	10 (3.9)	0.14 (0.06 to 0.29)		1.03 (0.37 to 2.83)	
Mozambique	45 (11.8)	31 (12.2)	0.59 (0.33 to 1.02)		1.42 (0.70 to 2.89)	
Sierra Leone	118 (31.0)	76 (29.8)	0.55 (0.36 to 0.84)		1.67 (0.93 to 3.02)	
South Africa	69 (18.1)	81 (31.8)	*Referent*		*Referent*	

* Excludes children wthat ho were dead on arrival or missing hospital duration, as well as unclassifiable diagnostic pairs (class VI).

† Bangladesh and Ethiopia were excluded from regression analysis due to low cell counts.

### Sensitivity analyses

The sensitivity and specificity of clinician antemortem diagnoses compared with postmortem causes of death varied by site (online supplemental table 5). However, test characteristics of antemortem diagnoses did not vary substantially by the sex (online supplemental table 6) or age of the deceased infant or child (online supplemental table 7). The sensitivity of antemortem clinician diagnoses was generally higher among infants and children who were admitted for ≥24 hours than those admitted for <24 hours prior to death. For instance, the sensitivity for lower respiratory tract infection in infants aged 1–11 months was 49.7% (95% CI 41.6% to 57.8%), for sepsis it was 47.7% (95% CI 39.4% to 56.0%) and for malnutrition it was 46.9% (95% CI 35.7% to 58.3%) (online supplemental table 8). Similar patterns were observed among those who had only one cause of death determined postmortem compared with those with more than one cause of death (online supplemental table 9). The sensitivity and specificity of clinical antemortem diagnoses made at the final clinical encounter were generally lower for cases that died at home than among cases that died during hospital admission (online supplemental table 10).

## Discussion

In our study of 881 deceased infants and children, we found that diagnostic errors were common when antemortem clinical diagnoses were compared with postmortem causes of death, which highlights the need for enhanced diagnostic approaches for young children. Many of the diagnostic discrepancies observed were major. Moreover, there were greater odds of antemortem clinical diagnostic errors among children who died from >1 cause. These findings underscore the limitations of clinical diagnosis and the critical need for improved diagnostic tools and techniques to reduce childhood mortality in resource-limited settings. Notably, many children died despite alignment between antemortem and postmortem diagnoses, underscoring that accurate diagnosis alone is insufficient to reduce childhood mortality but is a key step towards the provision of high-quality clinical care.

Approximately 40% of all deaths among infants and children in our study had at least one diagnostic error. This rate is higher than prior studies among deceased children that have demonstrated clinicopathological discrepancy rates ranging from 19% among children in Chile^11^ to 30% among children in China.^29^ The higher rate of clinicopathological discrepancies observed in our study compared with prior studies among children is likely due to several reasons. First, laboratory capabilities in low- and middle-income countries are often more limited than that found in settings with more resources,^7 30^ which may hinder the determination of timely and accurate antemortem diagnoses. Prior studies also suggest commonly used clinical signs and symptoms for diagnosing lower respiratory tract infections can be non-specific and difficult to detect correctly in young children, making accurate diagnosis of these conditions challenging.^31^ Accurate diagnoses among young children may also be more challenging given non-specific symptoms for many conditions, which has prompted the development of risk assessment tools that make use of combined signs and symptoms to augment diagnostic and prognostic accuracy,^32 33^ though these tools are not yet widely used.

Major diagnostic errors were more common among infants and children who had more than one cause of death. Children with more comorbid illnesses may have greater disease complexity that poses diagnostic challenges. Clinicians may prioritise diagnosing and treating one predominant condition while other comorbid illnesses are missed or not highlighted for treatment decisions. Additionally, overlapping signs and symptoms between concurrent diseases can complicate making accurate diagnoses. This finding may also represent underdocumentation of antemortem clinical diagnoses. However, we had no reason to believe that underdocumentation would be more common among children who died from >1 cause.

Clinician antemortem diagnosis of malaria had the highest sensitivity in our study. Owing to the historically large burden of malaria-related mortality, much attention has been paid to developing simple and accurate diagnostic tools including malaria rapid diagnostic tests and malaria parasite smears.^34^ However, the accuracy of malaria rapid diagnostic tests is limited by the persistence of positive results for weeks after successful treatment and cure. This may lead to clinician overdiagnosis of malaria by clinicians based on positive rapid diagnostic tests, even with resolved infection, contributing to the low positive predictive value of 40.6% we observed. Moreover, in malaria-endemic areas, individuals can be infected but not necessarily have any clinical consequence of those infections, given the partial natural immunity progressively acquired to this disease. The availability of such rapid diagnostics, as well as a high pretest probability in malaria-endemic settings, may have contributed to high sensitivity of antemortem clinician diagnosis. As the sensitivity of clinician antemortem diagnoses was low for the other included diseases, our results suggest that additional rapid, simple, accurate and clinically relevant diagnostics and point-of-care testing in settings with limited laboratory capacity may be needed to enhance diagnostic accuracy for conditions such as lower respiratory tract infections and sepsis.

The sensitivity of antemortem clinical diagnosis for common causes of death such as lower respiratory tract infections and sepsis was below 50%, rendering many of these diagnoses unrecognised while the child was alive. Moreover, the sensitivity of antemortem clinical diagnosis for other respiratory diseases (ie, aspiration pneumonia, interstitial lung disease, and pulmonary haemorrhage) was the lowest among the conditions considered. Potential factors contributing to this suboptimal diagnostic accuracy could include the lack of access to radiologic imaging, which may hinder the accurate identification of lung-related abnormalities.^35^ Additionally, the presence of non-specific signs and symptoms commonly associated with respiratory illnesses might further complicate precise diagnosis.

### Limitations

The results of our study should be interpreted in the context of several limitations. First, clinical data collected in the CHAMPS network relies on documentation by treating clinicians and nurses, which at times may be incomplete and may not have included all antemortem clinical diagnoses. Some diagnoses considered by treating clinicians may not have been documented, leading to a potential overestimation or underestimation, of clinicopathological discrepancies. Respiratory diseases, such as pneumonia, are often the terminal event preceding death. Therefore, symptoms on admission may not always translate to the cause of death determined postmortem. There is also the potential difficulty in differentiating between transient or asymptomatic bacteraemia/positive culture and sepsis or SIRS, as the determination of sepsis was based on culture sampling via MITS and required careful consideration of clinical context and symptoms, which may not always be clearly documented or distinguishable in antemortem records.

This discordance in symptoms at presentation through disease progression could contribute to the low sensitivity observed for other respiratory diseases in our analyses. We attempted to overcome these potential limitations by thorough review of all available clinical data by trained clinicians with familiarity with local contexts. We did not assess the relationship between the timing that the diagnosis was made and the postmortem-determined causes of death. As illnesses progress and patients can develop secondary illnesses, future studies assessing the concordance of antemortem and postmortem causes of death should include moderator analyses between the timing that antemortem diagnoses were made and postmortem causes of death. Furthermore, we did not assess whether diagnoses made by clinicians were for previously known chronic diseases (eg, chronic HIV disease) or new illnesses (eg, newly diagnosed HIV). Nevertheless, as our reference standard was robust postmortem determined causes of death that includes immediate, underlying and comorbid causes of death we do not expect this to have substantially affected our results. Some of the diagnoses considered discrepant, such as aspiration pneumonia, sepsis and interstitial lung disease, may reflect the natural progression of a primary illness rather than distinct, avertable causes of death, complicating the distinction between initial illness and subsequent conditions. Additionally, the diagnostic capabilities and skills of clinicians across sites vary and likely depend on the level of training of medical staff, which was not measured in this study. We did not separately analyse immediate, underlying and comorbid postmortem causes of death in relation to antemortem diagnoses. The classification system used to categorise major antemortem diagnostic errors does not necessarily indicate there was also an error in clinical management or treatment that could have prevented the child’s death. Some major errors may have still received appropriate supportive care, such as fluids, oxygen and antibiotics, which treat many conditions. Thus, while major diagnostic errors suggest potential opportunities to prevent mortality, it is difficult to quantify how many deaths were realistically avoidable. Some of the diagnoses considered discrepant, such as aspiration pneumonia, sepsis and interstitial lung disease, may reflect the natural progression of a primary illness rather than distinct, avertable causes of death, complicating the distinction between initial illness and subsequent conditions. We also did not capture the reasons clinicians made antemortem diagnoses or the use of equipment for diagnoses such as malnutrition through measurement of anthropometry, which warrants further investigation. Such an understanding may elucidate precise diagnostic challenges in need of targeted interventions to overcome. The relatively low specificity of antemortem diagnosis of diarrhoeal diseases could potentially be attributed to diarrhoea as a symptom accompanying other illnesses determined as the postmortem causes of death. Postmortem causes of death in CHAMPS are determined through a multifaceted approach that includes the integration of antemortem clinical diagnoses in addition to histopathological and microbiological MITS results. Lastly, our findings, although drawn from seven sites low-income and middle-income countries, may not represent the state of diagnostic accuracy in other settings in those countries or, more broadly, in sub-Saharan Africa and Bangladesh where postmortem examinations do not routinely occur.

## Conclusions

Antemortem clinical diagnostic errors were common among infants and children who died in seven regions in sub-Saharan Africa and Bangladesh with high childhood mortality rates. Comparing antemortem clinical diagnoses to postmortem causes of death may inform disease processes in most need of enhanced antemortem diagnostic approaches. Most antemortem clinical diagnostic errors were major. To further reduce childhood mortality in resource-limited settings, there is an urgent need to improve antemortem diagnostic capability through advances in availability of diagnostic testing and clinical skills.

## supplementary material

10.1136/bmjpo-2024-002654 online supplemental file 1

## Data Availability

Data are available on reasonable request.
