# Supplementary material for: Clinicopathological discrepancies in the diagnoses of childhood causes of death in the CHAMPS network: An analysis of antemortem diagnostic inaccuracies
Source: BMJ Paediatr Open. 2024 Jul 20;8(1):e002654. doi: 10.1136/bmjpo-2024-002654 (PMC11409330; doi:10.1136/bmjpo-2024-002654)
Supplement: online supplemental file 1 [file bmjpo-8-1-s001.pdf]

Supplemental Figure 1. Description of included cases of mortality among infants and children aged 1-59 months who died in one of seven sites in the Child Health and Mortality Prevention Surveillance Network

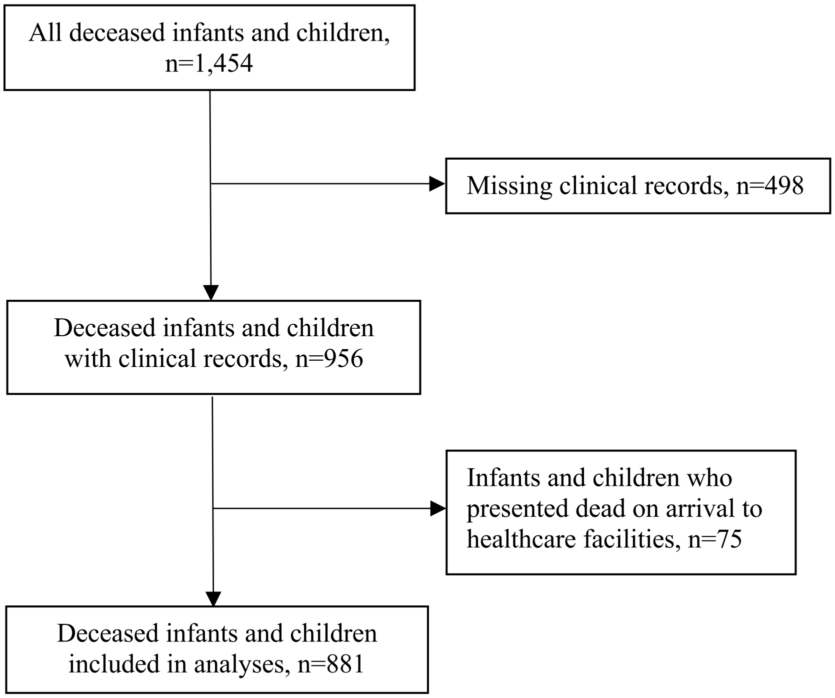

Supplemental Table 1. Comparison of included population (N=881) to those deaths in healthcare facilities with missing clinical records (N=498) who died from 2016-2022.

	**Included in Analyses, n (%)**	**Excluded due to missing clinical records, n (%)**	***P* value**
**Age in months (median, IQR)***	11 (4, 21)	11 (4, 24)	0.856
**Female**	417 (47.3)	220 (44.2)	0.261
**Site**			
Bangladesh	29 (3.3)	43 (8.6)	<0.001
Ethiopia	22 (2.5)	69 (13.9)	<0.001
Kenya	228 (25.9)	50 (10.0)	<0.001
Mali	135 (15.3)	100 (20.1)	0.025
Mozambique	88 (10.0)	184 (36.9)	<0.001
Sierra Leone	215 (24.4)	37 (7.4)	<0.001
South Africa	164 (18.6)	15 (3.0)	<0.001
**Most Common Causes of Death Anywhere in Causal Chain of Death**			
Lower respiratory tract infections	274 (31.1)	53 (10.6)	<0.001
Sepsis	258 (29.3)	35 (7.0)	<0.001
Malnutrition	167 (19.0)	27 (5.4)	<0.001
Malaria	142 (16.1)	12 (2.4)	<0.001
Diarrheal diseases	90 (10.2)	19 (3.8)	<0.001
HIV	68 (7.7)	8 (1.6)	<0.001
Congenital birth defects	63 (7.2)	6 (1.2)	<0.001
Anemias	93 (10.6)	2 (0.4)	<0.001
Other respiratory disease	50 (5.7)	4 (0.8)	<0.001
Meningitis/encephalitis	49 (5.6)	9 (1.8)	0.001

*Two cases had missing age but were categorized as infants or children at the time of death. *P*-values from Fisher's exact test and Kruskal–Wallis test.

Supplemental Table 2. Causes of death and location in causal chain among infants and children aged 1-59 months who died from 2016 to 2022

**Immediate Causes of Death Determined Postmortem**	**n (%)**
Sepsis	189 (21.5)
Lower respiratory infections	125 (14.2)
Malaria	49 (5.6)
Diarrheal diseases	30 (3.4)
Other respiratory disease	29 (3.3)
Anemias	29 (3.3)
Meningitis	13 (1.5)
Other infections	13 (1.5)
Liver disease	4 (0.5)
Other disorders of fluid, electrolyte, and acid-base balance	2 (0.2)
Poisoning	3 (0.3)
**Underlying Causes of Death Determined Postmortem**	
Malnutrition	119 (13.5)
Malaria	77 (8.7)
HIV	68 (7.7)
Lower respiratory infections	51 (5.8)
Congenital birth defects	58 (6.6)
Diarrheal disease	45 (5.1)
Sepsis	33 (3.7)
Neonatal preterm birth complications	33 (3.7)
Injury	23 (2.6)
Other infections	12 (1.4)
**Comorbid Causes of Death Determined Postmortem**	
Lower respiratory infections	110 (12.5)
Sepsis	58 (6.6)
Anemias	63 (7.2)
Malnutrition	47 (5.3)
Meningitis/encephalitis	34 (3.9)
Malaria	35 (4.0)
Diarrheal diseases	25 (2.8)
Other respiratory diseases	15 (1.7)
Other infections	21 (2.4)

Supplemental Table 3. Distribution of the antemortem clinical diagnoses and postmortem causes of death for N=881 deceased infants and children aged 1-59 months enrolled in CHAMPS sites from 2016 to 2022.

Clinical diagnoses	Postmortem Determined Cause of Death Anywhere in the Causal Chain of Death									
	**Lower respiratory tract infections, n (%)**	**Sepsis, n (%)**	**Malnutrition, n (%)**	**Malaria, n (%)**	**Diarrheal diseases, n (%)**	**HIV, n (%)**	**Congenital birth defects, n (%)**	**Anemias, n (%)**	**Other respiratory diseases, n (%)**	**Meningitis/ encephalitis, n (%)**
	N=274	N=258	N=167	N=142	N=90	N=68	N=63	N=93	N=50	N=49
Lower respiratory tract infection	130 (47.4)	72 (27.9)	53 (31.7)	28 (19.7)	20 (22.2)	31 (45.6)	30 (47.6)	25 (26.9)	22 (44.0)	13 (26.5)
Sepsis	84 (30.7)	96 (37.2)	26 (15.6)	9 (6.3)	15 (16.7)	17 (25.0)	26 (41.3)	10 (10.8)	12 (24.0)	21 (42.9)
Malnutrition	83 (30.3)	78 (30.2)	77 (46.1)	32 (22.5)	30 (33.3)	38 (55.9)	12 (19.0)	24 (25.8)	9 (18.0)	13 (26.5)
Malaria	53 (19.3)	45 (17.4)	64 (38.3)	114 (80.3)	23 (25.6)	17 (25.0)	6 (9.5)	64 (68.8)	14 (28.0)	2 (4.1)
Diarrheal diseases	90 (32.8)	88 (34.1)	65 (38.9)	38 (26.8)	74 (82.2)	27 (39.7)	11 (17.5)	24 (25.8)	17 (34.0)	7 (14.3)
HIV	36 (13.1)	20 (7.8)	6 (3.6)	11 (7.7)	7 (7.8)	45 (66.2)	0 (0)	5 (5.4)	4 (8.0)	4 (8.2)
Congenital birth defects	30 (10.9)	22 (8.5)	7 (4.2)	1 (0.7)	1 (1.1)	2 (2.9)	35 (55.6)	0 (0)	8 (16.0)	8 (16.3)
Anemias	48 (17.5)	53 (20.5)	36 (21.6)	37 (26.1)	14 (15.6)	19 (27.9)	6 (9.5)	34 (36.6)	10 (20.0)	8 (16.3)
Other respiratory diseases[1]	19 (6.9)	19 (7.4)	4 (2.4)	5 (3.5)	4 (4.4)	4 (5.9)	12 (19.0)	4 (4.3)	13 (26.0)	4 (8.2)
Meningitis/encephalitis	29 (10.6)	25 (9.7)	5 (3.0)	3 (2.1)	2 (2.2)	5 (7.4)	9 (14.3)	4 (4.3)	6 (12.0)	14 (28.6)

[1]Includes aspiration pneumonia, interstitial lung disease, and pulmonary hemorrhage

Supplemental Table 4. Performance of clinician antemortem diagnosis compared with postmortem causes of death among infants and children aged 1-59 months enrolled in CHAMPS sites from 2016 to 2022.

Postmortem Determined Cause of Death	Positive likelihood ratio (95% CI)	Negative likelihood ratio (95% CI)	Diagnostic odds ratio (95% CI)	Correctly classified proportion (95% CI)	Apparent prevalence (95% CI)	True prevalence (95% CI)	Youden's index (95% CI)	Number needed to diagnose (95% CI)
Lower respiratory tract infections	2.04 (1.69, 2.47)	0.68 (0.61, 0.77)	2.98 (2.20, 4.04)	0.677 (0.644, 0.707)	0.308 (0.277, 0.339)	0.311 (0.281, 0.343)	0.242 (0.146, 0.336)	4.1 (3.0, 6.8)
Sepsis	3.68 (2.77, 4.88)	0.70 (0.63, 0.77)	5.27 (3.67, 7.57)	0.745 (0.714, 0.773)	0.180 (0.156, 0.207)	0.293 (0.263, 0.324)	0.271 (0.185, 0.356)	3.7 (2.8, 5.4)
Malnutrition	2.70 (2.14, 3.40)	0.65 (0.56, 0.75)	4.15 (2.89, 5.96)	0.759 (0.730, 0.787)	0.226 (0.199, 0.255)	0.190 (0.164, 0.217)	0.290 (0.183, 0.396)	3.4 (2.5, 5.5)
Malaria	3.55 (3.04, 4.15)	0.25 (0.18, 0.36)	13.95 (8.91, 21.82)	0.779 (0.750, 0.806)	0.319 (0.288, 0.351)	0.161 (0.137, 0.187)	0.577 (0.470, 0.668)	1.7 (1.5, 2.1)
Diarrheal diseases	3.32 (2.84, 3.87)	0.24 (0.15, 0.37)	14.04 (7.99, 24.68)	0.759 (0.730, 0.787)	0.306 (0.276, 0.338)	0.102 (0.083, 0.124)	0.574 (0.448, 0.677)	1.7 (1.5, 2.2)
HIV	67.25 (33.06, 136.81)	0.34 (0.24, 0.48)	196.88 (83.42, 464.64)	0.965 (0.950, 0.976)	0.060 (0.045, 0.078)	0.077 (0.060, 0.097)	0.652 (0.518, 0.768)	1.5 (1.3, 1.9)
Congenital birth defects	9.88 (6.91, 14.12)	0.47 (0.36, 0.62)	20.98 (11.76, 37.44)	0.916 (0.896, 0.933)	0.092 (0.074, 0.113)	0.072 (0.055, 0.091)	0.499 (0.351, 0.639)	2.0 (1.6, 2.9)
Anemias	2.12 (1.56, 2.88)	0.77 (0.65, 0.90)	2.76 (1.74, 4.38)	0.779 (0.750, 0.806)	0.193 (0.167, 0.221)	0.106 (0.086, 0.128)	0.193 (0.067, 0.325)	5.2 (3.1, 14.9)
Other respiratory diseases*	6.35 (3.59, 11.26)	0.77 (0.65, 0.91)	8.24 (4.01, 16.91)	0.919 (0.899, 0.937)	0.053 (0.039, 0.070)	0.057 (0.042, 0.074)	0.219 (0.090, 0.375)	4.6 (2.7, 11.2)
Meningitis/encephalitis	4.40 (2.64, 7.35)	0.76 (0.64, 0.91)	5.76 (2.92, 11.36)	0.899 (0.877, 0.918)	0.077 (0.060, 0.097)	0.056 (0.041, 0.073)	0.221 (0.082, 0.383)	4.5 (2.6, 12.2)

*Includes aspiration pneumonia, interstitial lung disease, and pulmonary hemorrhage

Supplemental Table 5. Comparison of clinician antemortem diagnosis and postmortem causes of death among infants and children aged 1-59 months who died from 2016 to 2022 by CHAMPS site

Disease	True Positive, n (%)	True Negative, n (%)	False Positive, n (%)	False Negative, n (%)	Sensitivity % (95% CI)	Specificity % (95% CI)	Positive Predictive Value % (95% CI)	Negative Predictive Value % (95% CI)
Bangladesh, N=29								
Lower respiratory tract infections	0 (0)	21 (72.4)	7 (24.1)	1 (3.4)	0.0 (0.0, 97.5)	75.0 (55.1, 89.3)	0.0 (0.0, 41.0)	95.5 (77.2, 99.9)
Sepsis	2 (6.9)	25 (86.2)	1 (3.4)	1 (3.4)	66.7 (9.4, 99.2)	96.2 (80.4, 99.9)	66.7 (9.4, 99.2)	96.2 (80.4, 99.9)
Malnutrition	1 (3.4)	28 (96.6)	0 (0)	0 (0)	100.0 (2.5, 100.0)	100.0 (87.7, 100.0)	100.0 (2.5, 100.0)	100.0 (87.7, 100.0)
Malaria	0 (0)	29 (100.0)	0 (0)	0 (0)	--	100.0 (88.1, 100.0)	--	100.0 (88.1, 100.0)
Diarrheal diseases	1 (3.4)	27 (93.1)	1 (3.4)	0 (0)	100.0 (2.5, 100.0)	96.4 (81.7, 99.9)	50.0 (1.3, 98.7)	100.0 (87.2, 100.0)
HIV	0 (0)	29 (100.0)	0 (0)	0 (0)	--	100.0 (88.1, 100.0)	--	100.0 (88.1, 100.0)
Congenital birth defects	0 (0)	15 (51.7)	13 (44.8)	1 (3.4)	0.0 (0.0, 97.5)	53.6 (33.9, 72.5)	0.0 (0.0, 24.7)	93.8 (69.8, 99.8)
Anemias	0 (0)	26 (89.7)	2 (6.9)	1 (3.4)	0.0 (0.0, 97.5)	92.9 (76.5, 99.1)	0.0 (0.0, 84.2)	96.3 (81.0, 99.9)
Other respiratory disease*	0 (0)	29 (100.0)	0 (0)	0 (0)	--	100.0 (88.1, 100.0)	--	100.0 (88.1, 100.0)
Meningitis/encephalitis	0 (0)	29 (100.0)	0 (0)	0 (0)	--	100.0 (88.1, 100.0)	--	100.0 (88.1, 100.0)
Ethiopia, N=22								
Lower respiratory tract infection	4 (18.2)	13 (59.1)	2 (9.1)	3 (13.6)	57.1 (18.4, 90.1)	86.7 (59.5, 98.3)	66.7 (22.3, 95.7)	81.2 (54.4, 96.0)
Sepsis	2 (9.1)	11 (50.0)	1 (4.5)	8 (36.4)	20.0 (2.5, 55.6)	91.7 (61.5, 99.8)	66.7 (9.4, 99.2)	57.9 (33.5, 79.7)
Malnutrition	8 (36.4)	7 (31.8)	4 (18.2)	3 (13.6)	72.7 (39.0, 94.0)	63.6 (30.8, 89.1)	66.7 (34.9, 90.1)	70.0 (34.8, 93.3)
Malaria	0 (0)	22 (100.0)	0 (0)	0 (0)	--	100.0 (84.6, 100.0)	--	100.0 (84.6, 100.0)
Diarrheal diseases	3 (13.6)	14 (63.6)	2 (9.1)	3 (13.6)	50.0 (11.8, 88.2)	87.5 (61.7, 98.4)	60.0 (14.7, 94.7)	82.4 (56.6, 96.2)
HIV	0 (0)	22 (100.0)	0 (0)	0 (0)	--	100.0 (84.6, 100.0)	--	100.0 (84.6, 100.0)
Congenital birth defects	0 (0)	19 (86.4)	3 (13.6)	0 (0)	--	86.4 (65.1, 97.1)	0.0 (0.0, 70.8)	100.0 (82.4, 100.0)
Anemias	2 (9.1)	18 (81.8)	2 (9.1)	0 (0)	100.0 (15.8, 100.0)	90.0 (68.3, 98.8)	50.0 (6.8, 93.2)	100.0 (81.5, 100.0)
Other respiratory disease*	1 (4.5)	21 (95.5)	0 (0)	0 (0)	100.0 (2.5, 100.0)	100.0 (83.9, 100.0)	100.0 (2.5, 100.0)	100.0 (83.9, 100.0)
Meningitis/encephalitis	1 (4.5)	15 (68.2)	2 (9.1)	4 (18.2)	20.0 (0.5, 71.6)	88.2 (63.6, 98.5)	33.3 (0.8, 90.6)	78.9 (54.4, 93.9)
Kenya, N=228								
Lower respiratory tract infection	32 (14.0)	130 (57.0)	38 (16.7)	28 (12.3)	53.3 (40.0, 66.3)	77.4 (70.3, 83.5)	45.7 (33.7, 58.1)	82.3 (75.4, 87.9)
Sepsis	15 (6.6)	151 (66.2)	16 (7.0)	46 (20.2)	24.6 (14.5, 37.3)	90.4 (84.9, 94.4)	48.4 (30.2, 66.9)	76.6 (70.1, 82.4)
Malnutrition	33 (14.5)	140 (61.4)	18 (7.9)	37 (16.2)	47.1 (35.1, 59.4)	88.6 (82.6, 93.1)	64.7 (50.1, 77.6)	79.1 (72.4, 84.8)
Malaria	46 (20.2)	110 (48.2)	51 (22.4)	21 (9.2)	68.7 (56.2, 79.4)	68.3 (60.5, 75.4)	47.4 (37.2, 57.8)	84.0 (76.5, 89.8)
Diarrheal diseases	26 (11.4)	128 (56.1)	73 (32.0)	1 (0.4)	96.3 (81.0, 99.9)	63.7 (56.6, 70.3)	26.3 (17.9, 36.1)	99.2 (95.8, 100.0)
HIV	9 (3.9)	210 (92.1)	0 (0)	9 (3.9)	50.0 (26.0, 74.0)	100.0 (98.3, 100.0)	100.0 (66.4, 100.0)	95.9 (92.3, 98.1)
Congenital birth defects	7 (3.1)	209 (91.7)	5 (2.2)	7 (3.1)	50.0 (23.0, 77.0)	97.7 (94.6, 99.2)	58.3 (27.7, 84.8)	96.8 (93.4, 98.7)
Anemias	3 (1.3)	190 (83.3)	35 (15.4)	0 (0)	100.0 (29.2, 100.0)	84.4 (79.0, 88.9)	7.9 (1.7, 21.4)	100.0 (98.1, 100.0)
Other respiratory disease*	2 (0.9)	212 (93.0)	1 (0.4)	13 (5.7)	13.3 (1.7, 40.5)	99.5 (97.4, 100.0)	66.7 (9.4, 99.2)	94.2 (90.3, 96.9)
Meningitis/encephalitis	0 (0)	217 (95.2)	10 (4.4)	1 (0.4)	0.0 (0.0, 97.5)	95.6 (92.0, 97.9)	0.0 (0.0, 30.8)	99.5 (97.5, 100.0)
Mali, N=135								
Lower respiratory tract infection	4 (3.0)	88 (65.2)	29 (21.5)	14 (10.4)	22.2 (6.4, 47.6)	75.2 (66.4, 82.7)	12.1 (3.4, 28.2)	86.3 (78.0, 92.3)
Sepsis	3 (2.2)	109 (80.7)	5 (3.7)	18 (13.3)	14.3 (3.0, 36.3)	95.6 (90.1, 98.6)	37.5 (8.5, 75.5)	85.8 (78.5, 91.4)
Malnutrition	3 (2.2)	113 (83.7)	11 (8.1)	8 (5.9)	27.3 (6.0, 61.0)	91.1 (84.7, 95.5)	21.4 (4.7, 50.8)	93.4 (87.4, 97.1)
Malaria	2 (1.5)	111 (82.2)	22 (16.3)	0 (0)	100.0 (15.8, 100.0)	83.5 (76.0, 89.3)	8.3 (1.0, 27.0)	100.0 (96.7, 100.0)
Diarrheal diseases	4 (3.0)	102 (75.6)	27 (20.0)	2 (1.5)	66.7 (22.3, 95.7)	79.1 (71.0, 85.7)	12.9 (3.6, 29.8)	98.1 (93.2, 99.8)
HIV	0 (0)	131 (97.0)	0 (0)	4 (3.0)	0.0 (0.0, 60.2)	100.0 (97.2, 100.0)	--	97.0 (92.6, 99.2)
Congenital birth defects	2 (1.5)	120 (88.9)	10 (7.4)	3 (2.2)	40.0 (5.3, 85.3)	92.3 (86.3, 96.2)	16.7 (2.1, 48.4)	97.6 (93.0, 99.5)
Anemias	1 (0.7)	116 (85.9)	18 (13.3)	0 (0)	100.0 (2.5, 100.0)	86.6 (79.6, 91.8)	5.3 (0.1, 26.0)	100.0 (96.9, 100.0)

Other respiratory disease*	0 (0)	131 (97.0)	2 (1.5)	2 (1.5)	0.0 (0.0, 84.2)	98.5 (94.7, 99.8)	0.0 (0.0, 84.2)	98.5 (94.7, 99.8)
Meningitis/encephalitis	1 (0.7)	117 (86.7)	15 (11.1)	2 (1.5)	33.3 (0.8, 90.6)	88.6 (82.0, 93.5)	6.2 (0.2, 30.2)	98.3 (94.1, 99.8)
Mozambique, N=88								
Lower respiratory tract infection	19 (21.6)	43 (48.9)	9 (10.2)	17 (19.3)	52.8 (35.5, 69.6)	82.7 (69.7, 91.8)	67.9 (47.6, 84.1)	71.7 (58.6, 82.5)
Sepsis	7 (8.0)	53 (60.2)	11 (12.5)	17 (19.3)	29.2 (12.6, 51.1)	82.8 (71.3, 91.1)	38.9 (17.3, 64.3)	75.7 (64.0, 85.2)
Malnutrition	4 (4.5)	42 (47.7)	41 (46.6)	1 (1.1)	80.0 (28.4, 99.5)	50.6 (39.4, 61.8)	8.9 (2.5, 21.2)	97.7 (87.7, 99.9)
Malaria	17 (19.3)	61 (69.3)	10 (11.4)	0 (0)	100.0 (80.5, 100.0)	85.9 (75.6, 93.0)	63.0 (42.4, 80.6)	100.0 (94.1, 100.0)
Diarrheal diseases	16 (18.2)	48 (54.5)	22 (25.0)	2 (2.3)	88.9 (65.3, 98.6)	68.6 (56.4, 79.1)	42.1 (26.3, 59.2)	96.0 (86.3, 99.5)
HIV	16 (18.2)	66 (75.0)	3 (3.4)	3 (3.4)	84.2 (60.4, 96.6)	95.7 (87.8, 99.1)	84.2 (60.4, 96.6)	95.7 (87.8, 99.1)
Congenital birth defects	2 (2.3)	79 (89.8)	4 (4.5)	3 (3.4)	40.0 (5.3, 85.3)	95.2 (88.1, 98.7)	33.3 (4.3, 77.7)	96.3 (89.7, 99.2)
Anemias	4 (4.5)	63 (71.6)	20 (22.7)	1 (1.1)	80.0 (28.4, 99.5)	75.9 (65.3, 84.6)	16.7 (4.7, 37.4)	98.4 (91.6, 100.0)
Other respiratory disease*	3 (3.4)	69 (78.4)	11 (12.5)	5 (5.7)	37.5 (8.5, 75.5)	86.2 (76.7, 92.9)	21.4 (4.7, 50.8)	93.2 (84.9, 97.8)
Meningitis/encephalitis	1 (1.1)	85 (96.6)	2 (2.3)	0 (0)	100.0 (2.5, 100.0)	97.7 (91.9, 99.7)	33.3 (0.8, 90.6)	100.0 (95.8, 100.0)
Sierra Leone, N=215								
Lower respiratory tract infection	18 (8.4)	130 (60.5)	43 (20.0)	24 (11.2)	42.9 (27.7, 59.0)	75.1 (68.0, 81.4)	29.5 (18.5, 42.6)	84.4 (77.7, 89.8)
Sepsis	7 (3.3)	163 (75.8)	3 (1.4)	42 (19.5)	14.3 (5.9, 27.2)	98.2 (94.8, 99.6)	70.0 (34.8, 93.3)	79.5 (73.3, 84.8)
Malnutrition	22 (10.2)	131 (60.9)	23 (10.7)	39 (18.1)	36.1 (24.2, 49.4)	85.1 (78.4, 90.3)	48.9 (33.7, 64.2)	77.1 (70.0, 83.1)
Malaria	49 (22.8)	75 (34.9)	84 (39.1)	7 (3.3)	87.5 (75.9, 94.8)	47.2 (39.2, 55.2)	36.8 (28.6, 45.6)	91.5 (83.2, 96.5)
Diarrheal diseases	10 (4.7)	157 (73.0)	42 (19.5)	6 (2.8)	62.5 (35.4, 84.8)	78.9 (72.6, 84.3)	19.2 (9.6, 32.5)	96.3 (92.2, 98.6)
HIV	3 (1.4)	206 (95.8)	2 (0.9)	4 (1.9)	42.9 (9.9, 81.6)	99.0 (96.6, 99.9)	60.0 (14.7, 94.7)	98.1 (95.2, 99.5)
Congenital birth defects	0 (0)	215 (100.0)	0 (0)	0 (0)	--	100.0 (98.3, 100.0)	--	100.0 (98.3, 100.0)
Anemias	24 (11.2)	99 (46.0)	35 (16.3)	57 (26.5)	29.6 (20.0, 40.8)	73.9 (65.6, 81.1)	40.7 (28.1, 54.3)	63.5 (55.4, 71.0)
Other respiratory disease*	0 (0)	200 (93.0)	7 (3.3)	8 (3.7)	0.0 (0.0, 36.9)	96.6 (93.2, 98.6)	0.0 (0.0, 41.0)	96.2 (92.6, 98.3)
Meningitis/encephalitis	2 (0.9)	201 (93.5)	6 (2.8)	6 (2.8)	25.0 (3.2, 65.1)	97.1 (93.8, 98.9)	25.0 (3.2, 65.1)	97.1 (93.8, 98.9)
South Africa, N=164								
Lower respiratory tract infection	53 (32.3)	41 (25.0)	13 (7.9)	57 (34.8)	48.2 (38.6, 57.9)	75.9 (62.4, 86.5)	80.3 (68.7, 89.1)	41.8 (31.9, 52.2)
Sepsis	60 (36.6)	48 (29.3)	26 (15.9)	30 (18.3)	66.7 (55.9, 76.3)	64.9 (52.9, 75.6)	69.8 (58.9, 79.2)	61.5 (49.8, 72.3)
Malnutrition	6 (3.7)	131 (79.9)	25 (15.2)	2 (1.2)	75.0 (34.9, 96.8)	84.0 (77.3, 89.4)	19.4 (7.5, 37.5)	98.5 (94.7, 99.8)
Malaria	0 (0)	164 (100.0)	0 (0)	0 (0)	--	100.0 (97.8, 100.0)	--	100.0 (97.8, 100.0)
Diarrheal diseases	14 (8.5)	121 (73.8)	27 (16.5)	2 (1.2)	87.5 (61.7, 98.4)	81.8 (74.6, 87.6)	34.1 (20.1, 50.6)	98.4 (94.2, 99.8)
HIV	17 (10.4)	141 (86.0)	3 (1.8)	3 (1.8)	85.0 (62.1, 96.8)	97.9 (94.0, 99.6)	85.0 (62.1, 96.8)	97.9 (94.0, 99.6)
Congenital birth defects	24 (14.6)	115 (70.1)	11 (6.7)	14 (8.5)	63.2 (46.0, 78.2)	91.3 (84.9, 95.6)	68.6 (50.7, 83.1)	89.1 (82.5, 93.9)
Anemias	0 (0)	140 (85.4)	24 (14.6)	0 (0)	--	85.4 (79.0, 90.4)	0.0 (0.0, 14.2)	100.0 (97.4, 100.0)
Other respiratory disease*	7 (4.3)	135 (82.3)	13 (7.9)	9 (5.5)	43.8 (19.8, 70.1)	91.2 (85.4, 95.2)	35.0 (15.4, 59.2)	93.8 (88.5, 97.1)
Meningitis/encephalitis	9 (5.5)	114 (69.5)	19 (11.6)	22 (13.4)	29.0 (14.2, 48.0)	85.7 (78.6, 91.2)	32.1 (15.9, 52.4)	83.8 (76.5, 89.6)

*Includes aspiration pneumonia, interstitial lung disease, and pulmonary hemorrhage

Supplemental Table 6. Comparison of clinician antemortem diagnosis and postmortem causes of death among infants and children aged 1-59 months who died from 2016 to 2022 by sex

Disease	True Positive, n (%)	True Negative, n (%)	False Positive, n (%)	False Negative, n (%)	Sensitivity % (95% CI)	Specificity % (95% CI)	Positive Predictive Value % (95% CI)	Negative Predictive Value % (95% CI)
Females, N=417								
Lower respiratory tract infection	64 (15.3)	222 (53.2)	69 (16.5)	62 (14.9)	50.8 (41.7, 59.8)	76.3 (71.0, 81.1)	48.1 (39.4, 56.9)	78.2 (72.9, 82.8)
Sepsis	44 (10.6)	261 (62.6)	31 (7.4)	81 (19.4)	35.2 (26.9, 44.2)	89.4 (85.3, 92.7)	58.7 (46.7, 69.9)	76.3 (71.4, 80.7)
Malnutrition	37 (8.9)	292 (70.0)	50 (12.0)	38 (9.1)	49.3 (37.6, 61.1)	85.4 (81.2, 89.0)	42.5 (32.0, 53.6)	88.5 (84.5, 91.7)
Malaria	57 (13.7)	277 (66.4)	70 (16.8)	13 (3.1)	81.4 (70.3, 89.7)	79.8 (75.2, 83.9)	44.9 (36.1, 54.0)	95.5 (92.5, 97.6)
Diarrheal diseases	41 (9.8)	273 (65.5)	99 (23.7)	4 (1.0)	91.1 (78.8, 97.5)	73.4 (68.6, 77.8)	29.3 (21.9, 37.6)	98.6 (96.3, 99.6)
HIV	21 (5.0)	377 (90.4)	4 (1.0)	15 (3.6)	58.3 (40.8, 74.5)	99.0 (97.3, 99.7)	84.0 (63.9, 95.5)	96.2 (93.8, 97.8)
Congenital birth defects	17 (4.1)	361 (86.6)	26 (6.2)	13 (3.1)	56.7 (37.4, 74.5)	93.3 (90.3, 95.6)	39.5 (25.0, 55.6)	96.5 (94.1, 98.1)
Anemias	15 (3.6)	299 (71.7)	69 (16.5)	34 (8.2)	30.6 (18.3, 45.4)	81.2 (76.9, 85.1)	17.9 (10.4, 27.7)	89.8 (86.0, 92.8)
Other respiratory disease*	3 (0.7)	378 (90.6)	17 (4.1)	19 (4.6)	13.6 (2.9, 34.9)	95.7 (93.2, 97.5)	15.0 (3.2, 37.9)	95.2 (92.6, 97.1)
Meningitis/encephalitis	2 (0.5)	373 (89.4)	28 (6.7)	14 (3.4)	12.5 (1.6, 38.3)	93.0 (90.1, 95.3)	6.7 (0.8, 22.1)	96.4 (94.0, 98.0)
Males, N=464								
Lower respiratory tract infection	66 (14.2)	244 (52.6)	72 (15.5)	82 (17.7)	44.6 (36.4, 53.0)	77.2 (72.2, 81.7)	47.8 (39.3, 56.5)	74.8 (69.8, 79.5)
Sepsis	52 (11.2)	299 (64.4)	32 (6.9)	81 (17.5)	39.1 (30.8, 47.9)	90.3 (86.6, 93.3)	61.9 (50.7, 72.3)	78.7 (74.2, 82.7)
Malnutrition	40 (8.6)	300 (64.7)	72 (15.5)	52 (11.2)	43.5 (33.2, 54.2)	80.6 (76.3, 84.5)	35.7 (26.9, 45.3)	85.2 (81.1, 88.8)
Malaria	57 (12.3)	295 (63.6)	97 (20.9)	15 (3.2)	79.2 (68.0, 87.8)	75.3 (70.7, 79.4)	37.0 (29.4, 45.2)	95.2 (92.1, 97.3)
Diarrheal diseases	33 (7.1)	322 (69.4)	97 (20.9)	12 (2.6)	73.3 (58.1, 85.4)	76.8 (72.5, 80.8)	25.4 (18.2, 33.8)	96.4 (93.8, 98.1)
HIV	24 (5.2)	428 (92.2)	4 (0.9)	8 (1.7)	75.0 (56.6, 88.5)	99.1 (97.6, 99.7)	85.7 (67.3, 96.0)	98.2 (96.4, 99.2)
Congenital birth defects	18 (3.9)	411 (88.6)	20 (4.3)	15 (3.2)	54.5 (36.4, 71.9)	95.4 (92.9, 97.1)	47.4 (31.0, 64.2)	96.5 (94.3, 98.0)
Anemias	19 (4.1)	353 (76.1)	67 (14.4)	25 (5.4)	43.2 (28.3, 59.0)	84.0 (80.2, 87.4)	22.1 (13.9, 32.3)	93.4 (90.4, 95.7)
Other respiratory disease*	10 (2.2)	419 (90.3)	17 (3.7)	18 (3.9)	35.7 (18.6, 55.9)	96.1 (93.8, 97.7)	37.0 (19.4, 57.6)	95.9 (93.6, 97.5)
Meningitis/encephalitis	12 (2.6)	405 (87.3)	26 (5.6)	21 (4.5)	36.4 (20.4, 54.9)	94.0 (91.3, 96.0)	31.6 (17.5, 48.7)	95.1 (92.6, 96.9)

*Includes aspiration pneumonia, interstitial lung disease, and pulmonary hemorrhage

Supplemental Table 7. Comparison of clinician antemortem diagnosis and postmortem causes of death among infants and children aged 1-59 months who died from 2016 to 2022 by age

**Disease**	**True Positive, n (%)**	**True Negative, n (%)**	**False Positive, n (%)**	**False Negative, n (%)**	**Sensitivity % (95% CI)**	**Specificity % (95% CI)**	**Positive Predictive Value % (95% CI)**	**Negative Predictive Value % (95% CI)**
Infants aged 1-11 months, N=465								
Lower respiratory tract infection	78 (16.8)	227 (48.8)	81 (17.4)	79 (17.0)	49.7 (41.6, 57.8)	73.7 (68.4, 78.5)	49.1 (41.1, 57.1)	74.2 (68.9, 79.0)
Sepsis	71 (15.3)	276 (59.4)	40 (8.6)	78 (16.8)	47.7 (39.4, 56.0)	87.3 (83.2, 90.8)	64.0 (54.3, 72.9)	78.0 (73.3, 82.2)
Malnutrition	38 (8.2)	332 (71.4)	52 (11.2)	43 (9.2)	46.9 (35.7, 58.3)	86.5 (82.6, 89.7)	42.2 (31.9, 53.1)	88.5 (84.9, 91.6)
Malaria	35 (7.5)	359 (77.2)	62 (13.3)	9 (1.9)	79.5 (64.7, 90.2)	85.3 (81.5, 88.5)	36.1 (26.6, 46.5)	97.6 (95.4, 98.9)
Diarrheal diseases	44 (9.5)	315 (67.7)	102 (21.9)	4 (0.9)	91.7 (80.0, 97.7)	75.5 (71.1, 79.6)	30.1 (22.8, 38.3)	98.7 (96.8, 99.7)
HIV	18 (3.9)	429 (92.3)	6 (1.3)	12 (2.6)	60.0 (40.6, 77.3)	98.6 (97.0, 99.5)	75.0 (53.3, 90.2)	97.3 (95.3, 98.6)
Congenital birth defects	26 (5.6)	383 (82.4)	37 (8.0)	19 (4.1)	57.8 (42.2, 72.3)	91.2 (88.1, 93.7)	41.3 (29.0, 54.4)	95.3 (92.7, 97.1)
Anemias	8 (1.7)	375 (80.6)	64 (13.8)	18 (3.9)	30.8 (14.3, 51.8)	85.4 (81.8, 88.6)	11.1 (4.9, 20.7)	95.4 (92.9, 97.3)
Other respiratory disease*	5 (1.1)	424 (91.2)	20 (4.3)	16 (3.4)	23.8 (8.2, 47.2)	95.5 (93.1, 97.2)	20.0 (6.8, 40.7)	96.4 (94.2, 97.9)
Meningitis/encephalitis	12 (2.6)	399 (85.8)	33 (7.1)	21 (4.5)	36.4 (20.4, 54.9)	92.4 (89.4, 94.7)	26.7 (14.6, 41.9)	95.0 (92.5, 96.9)
Children aged 12-59 months, N=464								
Lower respiratory tract infection	52 (12.5)	239 (57.5)	60 (14.4)	65 (15.6)	44.4 (35.3, 53.9)	79.9 (74.9, 84.3)	46.4 (37.0, 56.1)	78.6 (73.6, 83.1)
Sepsis	25 (6.0)	284 (68.3)	23 (5.5)	84 (20.2)	22.9 (15.4, 32.0)	92.5 (89.0, 95.2)	52.1 (37.2, 66.7)	77.2 (72.5, 81.4)
Malnutrition	39 (9.4)	260 (62.5)	70 (16.8)	47 (11.3)	45.3 (34.6, 56.5)	78.8 (74.0, 83.1)	35.8 (26.8, 45.5)	84.7 (80.2, 88.5)
Malaria	79 (19.0)	213 (51.2)	105 (25.2)	19 (4.6)	80.6 (71.4, 87.9)	67.0 (61.5, 72.1)	42.9 (35.7, 50.4)	91.8 (87.5, 95.0)
Diarrheal diseases	30 (7.2)	280 (67.3)	94 (22.6)	12 (2.9)	71.4 (55.4, 84.3)	74.9 (70.1, 79.2)	24.2 (17.0, 32.7)	95.9 (92.9, 97.9)
HIV	27 (6.5)	376 (90.4)	2 (0.5)	11 (2.6)	71.1 (54.1, 84.6)	99.5 (98.1, 99.9)	93.1 (77.2, 99.2)	97.2 (95.0, 98.6)
Congenital birth defects	9 (2.2)	389 (93.5)	9 (2.2)	9 (2.2)	50.0 (26.0, 74.0)	97.7 (95.8, 99.0)	50.0 (26.0, 74.0)	97.7 (95.8, 99.0)
Anemias	26 (6.2)	277 (66.6)	72 (17.3)	41 (9.9)	38.8 (27.1, 51.5)	79.4 (74.7, 83.5)	26.5 (18.1, 36.4)	87.1 (82.9, 90.6)
Other respiratory disease*	8 (1.9)	373 (89.7)	14 (3.4)	21 (5.0)	27.6 (12.7, 47.2)	96.4 (94.0, 98.0)	36.4 (17.2, 59.3)	94.7 (92.0, 96.7)
Meningitis/encephalitis	2 (0.5)	379 (91.1)	21 (5.0)	14 (3.4)	12.5 (1.6, 38.3)	94.8 (92.1, 96.7)	8.7 (1.1, 28.0)	96.4 (94.1, 98.0)

*Includes aspiration pneumonia, interstitial lung disease, and pulmonary hemorrhage

Supplemental Table 8. Comparison of clinician antemortem diagnosis and postmortem causes of death among infants and children aged 1-59 months who died from 2016 to 2022 by hospital admission duration

**Disease**	**True Positive, n (%)**	**True Negative, n (%)**	**False Positive, n (%)**	**False Negative, n (%)**	**Sensitivity % (95% CI)**	**Specificity % (95% CI)**	**Positive Predictive Value % (95% CI)**	**Negative Predictive Value % (95% CI)**
<24 hours hospital admission before death, N=500								
Lower respiratory tract infection	44 (8.8)	305 (61.0)	80 (16.0)	71 (14.2)	38.3 (29.4, 47.8)	79.2 (74.8, 83.2)	35.5 (27.1, 44.6)	81.1 (76.8, 84.9)
Sepsis	19 (3.8)	365 (73.0)	25 (5.0)	91 (18.2)	17.3 (10.7, 25.7)	93.6 (90.7, 95.8)	43.2 (28.3, 59.0)	80.0 (76.1, 83.6)
Malnutrition	34 (6.8)	368 (73.6)	39 (7.8)	59 (11.8)	36.6 (26.8, 47.2)	90.4 (87.1, 93.1)	46.6 (34.8, 58.6)	86.2 (82.5, 89.3)
Malaria	75 (15.0)	294 (58.8)	110 (22.0)	21 (4.2)	78.1 (68.5, 85.9)	72.8 (68.2, 77.1)	40.5 (33.4, 48.0)	93.3 (90.0, 95.8)
Diarrheal diseases	43 (8.6)	352 (70.4)	98 (19.6)	7 (1.4)	86.0 (73.3, 94.2)	78.2 (74.1, 82.0)	30.5 (23.0, 38.8)	98.1 (96.0, 99.2)
HIV	10 (2.0)	475 (95.0)	2 (0.4)	13 (2.6)	43.5 (23.2, 65.5)	99.6 (98.5, 99.9)	83.3 (51.6, 97.9)	97.3 (95.5, 98.6)
Congenital birth defects	10 (2.0)	457 (91.4)	22 (4.4)	11 (2.2)	47.6 (25.7, 70.2)	95.4 (93.1, 97.1)	31.2 (16.1, 50.0)	97.6 (95.8, 98.8)
Anemias	22 (4.4)	377 (75.4)	75 (15.0)	26 (5.2)	45.8 (31.4, 60.8)	83.4 (79.7, 86.7)	22.7 (14.8, 32.3)	93.5 (90.7, 95.7)
Other respiratory disease*	2 (0.4)	464 (92.8)	10 (2.0)	24 (4.8)	7.7 (0.9, 25.1)	97.9 (96.2, 99.0)	16.7 (2.1, 48.4)	95.1 (92.8, 96.8)
Meningitis/encephalitis	6 (1.2)	461 (92.2)	23 (4.6)	10 (2.0)	37.5 (15.2, 64.6)	95.2 (93.0, 97.0)	20.7 (8.0, 39.7)	97.9 (96.1, 99.0)
≥24 hours hospital admission before death, N=381								
Lower respiratory tract infection	86 (22.6)	161 (42.3)	61 (16.0)	73 (19.2)	54.1 (46.0, 62.0)	72.5 (66.1, 78.3)	58.5 (50.1, 66.6)	68.8 (62.4, 74.7)
Sepsis	77 (20.2)	195 (51.2)	38 (10.0)	71 (18.6)	52.0 (43.7, 60.3)	83.7 (78.3, 88.2)	67.0 (57.6, 75.4)	73.3 (67.6, 78.5)
Malnutrition	43 (11.3)	224 (58.8)	83 (21.8)	31 (8.1)	58.1 (46.1, 69.5)	73.0 (67.6, 77.9)	34.1 (25.9, 43.1)	87.8 (83.2, 91.6)
Malaria	39 (10.2)	278 (73.0)	57 (15.0)	7 (1.8)	84.8 (71.1, 93.7)	83.0 (78.5, 86.9)	40.6 (30.7, 51.1)	97.5 (95.0, 99.0)
Diarrheal diseases	31 (8.1)	243 (63.8)	98 (25.7)	9 (2.4)	77.5 (61.5, 89.2)	71.3 (66.1, 76.0)	24.0 (16.9, 32.3)	96.4 (93.3, 98.4)
HIV	35 (9.2)	330 (86.6)	6 (1.6)	10 (2.6)	77.8 (62.9, 88.8)	98.2 (96.2, 99.3)	85.4 (70.8, 94.4)	97.1 (94.7, 98.6)
Congenital birth defects	25 (6.6)	315 (82.7)	24 (6.3)	17 (4.5)	59.5 (43.3, 74.4)	92.9 (89.6, 95.4)	51.0 (36.3, 65.6)	94.9 (91.9, 97.0)
Anemias	12 (3.1)	275 (72.2)	61 (16.0)	33 (8.7)	26.7 (14.6, 41.9)	81.8 (77.3, 85.8)	16.4 (8.8, 27.0)	89.3 (85.3, 92.5)
Other respiratory disease*	11 (2.9)	333 (87.4)	24 (6.3)	13 (3.4)	45.8 (25.6, 67.2)	93.3 (90.2, 95.6)	31.4 (16.9, 49.3)	96.2 (93.7, 98.0)
Meningitis/encephalitis	8 (2.1)	317 (83.2)	31 (8.1)	25 (6.6)	24.2 (11.1, 42.3)	91.1 (87.6, 93.9)	20.5 (9.3, 36.5)	92.7 (89.4, 95.2)

*Includes aspiration pneumonia, interstitial lung disease, and pulmonary hemorrhage

Supplemental Table 9. Comparison of clinician antemortem diagnosis and postmortem causes of death among infants and children aged 1-59 months who died from 2016 to 2022 by number of causes of death

Disease	True Positive, n (%)	True Negative, n (%)	False Positive, n (%)	False Negative, n (%)	Sensitivity % (95% CI)	Specificity % (95% CI)	Positive Predictive Value % (95% CI)	Negative Predictive Value % (95% CI)
One cause of death, N=115								
Lower respiratory tract infection	16 (13.9)	75 (65.2)	18 (15.7)	6 (5.2)	72.7 (49.8, 89.3)	80.6 (71.1, 88.1)	47.1 (29.8, 64.9)	92.6 (84.6, 97.2)
Sepsis	0 (0)	93 (80.9)	13 (11.3)	9 (7.8)	0.0 (0.0, 33.6)	87.7 (79.9, 93.3)	0.0 (0.0, 24.7)	91.2 (83.9, 95.9)
Malnutrition	3 (2.6)	100 (87.0)	12 (10.4)	0 (0)	100.0 (29.2, 100.0)	89.3 (82.0, 94.3)	20.0 (4.3, 48.1)	100.0 (96.4, 100.0)
Malaria	38 (33.0)	56 (48.7)	16 (13.9)	5 (4.3)	88.4 (74.9, 96.1)	77.8 (66.4, 86.7)	70.4 (56.4, 82.0)	91.8 (81.9, 97.3)
Diarrheal diseases	16 (13.9)	73 (63.5)	25 (21.7)	1 (0.9)	94.1 (71.3, 99.9)	74.5 (64.7, 82.8)	39.0 (24.2, 55.5)	98.6 (92.7, 100.0)
HIV	0 (0)	114 (99.1)	0 (0)	1 (0.9)	0.0 (0.0, 97.5)	100.0 (96.8, 100.0)	--	99.1 (95.3, 100.0)
Congenital birth defects	1 (0.9)	112 (97.4)	1 (0.9)	1 (0.9)	50.0 (1.3, 98.7)	99.1 (95.2, 100.0)	50.0 (1.3, 98.7)	99.1 (95.2, 100.0)
Anemias	0 (0)	95 (82.6)	20 (17.4)	0 (0)	--	82.6 (74.4, 89.0)	0.0 (0.0, 16.8)	100.0 (96.2, 100.0)
Other respiratory disease*	2 (1.7)	104 (90.4)	6 (5.2)	3 (2.6)	40.0 (5.3, 85.3)	94.5 (88.5, 98.0)	25.0 (3.2, 65.1)	97.2 (92.0, 99.4)
Meningitis/encephalitis	0 (0)	108 (93.9)	7 (6.1)	0 (0)	--	93.9 (87.9, 97.5)	0.0 (0.0, 41.0)	100.0 (96.6, 100.0)
More than one cause of death, N=470								
Lower respiratory tract infection	114 (24.3)	172 (36.6)	46 (9.8)	138 (29.4)	45.2 (39.0, 51.6)	78.9 (72.9, 84.1)	71.2 (63.6, 78.1)	55.5 (49.8, 61.1)
Sepsis	96 (20.4)	187 (39.8)	34 (7.2)	153 (32.6)	38.6 (32.5, 44.9)	84.6 (79.2, 89.1)	73.8 (65.4, 81.2)	55.0 (49.5, 60.4)
Malnutrition	74 (15.7)	238 (50.6)	68 (14.5)	90 (19.1)	45.1 (37.4, 53.1)	77.8 (72.7, 82.3)	52.1 (43.6, 60.6)	72.6 (67.4, 77.3)
Malaria	76 (16.2)	321 (68.3)	50 (10.6)	23 (4.9)	76.8 (67.2, 84.7)	86.5 (82.6, 89.8)	60.3 (51.2, 68.9)	93.3 (90.1, 95.7)
Diarrheal diseases	58 (12.3)	302 (64.3)	95 (20.2)	15 (3.2)	79.5 (68.4, 88.0)	76.1 (71.6, 80.2)	37.9 (30.2, 46.1)	95.3 (92.3, 97.3)
HIV	45 (9.6)	397 (84.5)	6 (1.3)	22 (4.7)	67.2 (54.6, 78.2)	98.5 (96.8, 99.5)	88.2 (76.1, 95.6)	94.7 (92.2, 96.7)
Congenital birth defects	34 (7.2)	395 (84.0)	14 (3.0)	27 (5.7)	55.7 (42.4, 68.5)	96.6 (94.3, 98.1)	70.8 (55.9, 83.0)	93.6 (90.8, 95.7)
Anemias	34 (7.2)	319 (67.9)	58 (12.3)	59 (12.6)	36.6 (26.8, 47.2)	84.6 (80.6, 88.1)	37.0 (27.1, 47.7)	84.4 (80.3, 87.9)
Other respiratory disease*	11 (2.3)	403 (85.7)	22 (4.7)	34 (7.2)	24.4 (12.9, 39.5)	94.8 (92.3, 96.7)	33.3 (18.0, 51.8)	92.2 (89.3, 94.6)
Meningitis/encephalitis	14 (3.0)	394 (83.8)	27 (5.7)	35 (7.4)	28.6 (16.6, 43.3)	93.6 (90.8, 95.7)	34.1 (20.1, 50.6)	91.8 (88.8, 94.3)

*Includes aspiration pneumonia, interstitial lung disease, and pulmonary hemorrhage

Supplemental Table 10. Comparison of clinician antemortem diagnosis and postmortem causes of death among infants and children aged 1-59 months who died at home following clinical encounters from 2016 to 2022

	**True Positive, n (%)**	**True Negative, n (%)**	**False Positive, n (%)**	**False Negative, n (%)**	**Sensitivity % (95% CI)**	**Specificity % (95% CI)**	**Positive Predictive Value % (95% CI)**	**Negative Predictive Value % (95% CI)**
Lower respiratory tract infection	14 (6.2)	147 (65.6)	34 (15.2)	29 (12.9)	32.6 (19.1, 48.5)	81.2 (74.8, 86.6)	29.2 (17.0, 44.1)	83.5 (77.2, 88.7)
Sepsis	4 (1.8)	170 (75.9)	9 (4.0)	41 (18.3)	8.9 (2.5, 21.2)	95.0 (90.7, 97.7)	30.8 (9.1, 61.4)	80.6 (74.6, 85.7)
Malnutrition	14 (6.2)	163 (72.8)	17 (7.6)	30 (13.4)	31.8 (18.6, 47.6)	90.6 (85.3, 94.4)	45.2 (27.3, 64.0)	84.5 (78.6, 89.3)
Malaria	21 (9.4)	158 (70.5)	33 (14.7)	12 (5.4)	63.6 (45.1, 79.6)	82.7 (76.6, 87.8)	38.9 (25.9, 53.1)	92.9 (88.0, 96.3)
Diarrheal diseases	11 (4.9)	169 (75.4)	42 (18.8)	2 (0.9)	84.6 (54.6, 98.1)	80.1 (74.1, 85.3)	20.8 (10.8, 34.1)	98.8 (95.8, 99.9)
HIV	1 (0.4)	213 (95.1)	1 (0.4)	9 (4.0)	10.0 (0.3, 44.5)	99.5 (97.4, 100.0)	50.0 (1.3, 98.7)	95.9 (92.4, 98.1)
Congenital birth defects	4 (1.8)	201 (89.7)	16 (7.1)	3 (1.3)	57.1 (18.4, 90.1)	92.6 (88.3, 95.7)	20.0 (5.7, 43.7)	98.5 (95.8, 99.7)
Anemias	2 (0.9)	199 (88.8)	23 (10.3)	0 (0)	100.0 (15.8, 100.0)	89.6 (84.9, 93.3)	8.0 (1.0, 26.0)	100.0 (98.2, 100.0)
Other respiratory diseases*	3 (1.3)	212 (94.6)	3 (1.3)	6 (2.7)	33.3 (7.5, 70.1)	98.6 (96.0, 99.7)	50.0 (11.8, 88.2)	97.2 (94.1, 99.0)
Meningitis/encephalitis	1 (0.4)	211 (94.2)	7 (3.1)	5 (2.2)	16.7 (0.4, 64.1)	96.8 (93.5, 98.7)	12.5 (0.3, 52.7)	97.7 (94.7, 99.2)

*Includes aspiration pneumonia, interstitial lung disease, and pulmonary hemorrhage
