# Supplementary material for: Clinicopathological discrepancies in the diagnoses of childhood causes of death in the CHAMPS network: An analysis of antemortem diagnostic inaccuracies
Source: BMJ Paediatr Open. 2024 Jul 20;8(1):e002654. doi: 10.1136/bmjpo-2024-002654 (PMC11409330; doi:10.1136/bmjpo-2024-002654)
Supplement: Supplementary file 2 [file bmjpo-2024-002654.reviewer_comments.pdf]

PEER REVIEW HISTORY

BMJ Paediatrics Open publishes all reviews undertaken for accepted manuscripts. Reviewers are asked to complete a checklist review form and are provided with free text boxes to elaborate on their assessment. These free text comments are reproduced below.

ARTICLE DETAILS

**TITLE (PROVISIONAL)**	Clinicopathological discrepancies in the diagnoses of childhood causes of death in the CHAMPS network: An analysis of antemortem diagnostic inaccuracies
**AUTHORS**	Leulseged, Haleluya<br>Bethencourt, Christine<br>Igunza, Kitiezo<br>Onyango, Dickens<br>Akelo, Victor<br>Omore, Richard<br>Ogbuanu, Ikechukwu<br>Ameh, Soter<br>Moseray, Andrew<br>Kowuor, Dickens<br>Bassey, Ima-Abasi<br>El Arifeen, Shams<br>Gurley, Emily S.<br>Hossain, Mohammad Zahid<br>Rahman, Afruna<br>Alam, Muntasir<br>Assefa, Nega<br>Madrid, Lola<br>Alemu, Addisu<br>Abdullahi, Yasir<br>Kotloff, Karen<br>Sow, Samba O.<br>Tapia, Milagritos<br>Kourouma, Nana<br>Sissoko, Seydou<br>Bassat, Quique<br>Varo, Rosauro<br>Mandomando, Inacio<br>Carrilho, Carla<br>Rakislova, Natalia<br>Fernandes, Fabiola<br>Madhi, Shabir<br>Dangor, Ziyaad<br>Mahtab, Sana<br>Hale, Martin<br>Baillie, Vicky<br>du Toit, Jeanie<br>Madewell, Zachary<br>Blau, Dianna<br>Martines, Roosecelis<br>Mutevedzi, Portia C.<br>Breiman, Robert<br>Whitney, Cynthia<br>Rees, Chris

VERSION 1 – REVIEW

**REVIEWER**	Dr. Judd L. Walson<br>Childhood Acute Illness<br>9602 Marmount Dr NW<br>Seattle<br>98117<br>United States
**REVIEW RETURNED**	15-Apr-2024

**GENERAL COMMENTS**	This is a very interesting manuscript that attempts to compare antenatal diagnoses in children in the CHAMPS network with postmortem ascertainment of COD. The results are of clinical and public health interest and suggest that significant discrepancies in antemortem and postmortem assessment of cause of illness may exist. The CHAMPS network has used innovative approaches to address COD that provide useful information to guide public health decision making in LMIC settings and these data add to that rich knowledge base.<br><br>There are some issues that should be addressed in the manuscript in order to help clinicians and public health professionals/poiicy makers interpret these findings.<br><br>First, it is important to note that several studies in settings similar to CHAMPS have now shown that as much as 50% of all mortality happens after hospital discharge and that causes of these later deaths may be different from those that led to the acute illness event that prompted initial hospitalization. This finding suggests that the important target of intervention in acutely ill children in these settings is not the disease or syndrome, but the child. The finding that different postmortem causes of disease are present may simply reflect the evolution of disease processes in a susceptible host and not be identifiable at the time of admission. Of course, some missed diagnoses could have large clinical impact, and identifying children in whom a correct diagnosis would have been treated differently could have impact. In order to determine how much of the misclassified diagnoses were potentially avertable, there are several considerations that should be addressed. First, it is critical to understand the timing between antemortem and postmortem diagnosis. The longer this time is, the greater the likelihood that this may represent a new onset of disease and not simply a missed diagnosis. The authors should present data regarding these timing issues and discuss whether time is an effect modifier in these analyses.<br><br>In addition, it is important to note if these diagnoses were carry over diagnoses from prior admissions or health care encounters or reflect new diagnoses made by clinicians based on laboratory or clinical assessment at the time of evaluation (for example, a clinician may assign HIV as a cause given knowledge of the childs HIV status from a prior hospitalization – that is different than making the diagnosis of an HIV associated complication at the time of the encounter).<br><br>The authors also apparently considered each cause of death to be a match if the antemortem clinical diagnosis was in any location in the causal chain. From a clinical or public health standpoint, this is difficult to interpret. Unless all potential causes of death are correctly ascertained and managed, it is unlikely that these deaths would have been averted. It would be useful to present analyses in which

	the matching required that all postmortem identified causes were included in the antemortem diagnosis.<br><br>Finally, some of the diagnoses that were considered discrepant (aspiration pneumonia, sepsis, interstitial lung disease, etc) may simply reflect a worsening clinical process following an illness (lower respiratory infection leading to bacteremia, sepsis leading to obtundation and then aspiration pneumonia) and may not reflect separate avertable causes of death – they may reflect that the patient is dying of the first correctly ascertained cause.<br><br>Overall, this is a useful manuscript that will add to the existing literature.

**REVIEWER**	Dr. Pui-Ying Iroh Tam<br>Malawi-Liverpool-Wellcome Trust Clinical Research Programme<br>P.O. Box 30096<br>Blantyre<br>Malawi
**REVIEW RETURNED**	10-May-2024

**GENERAL COMMENTS**	General comments: Important topic on accuracy of clinical diagnoses in young children who have died, in relation to specific histopathological and microbiological testing of biosamples obtained as a result of CHAMPS. In low resource settings with few diagnostics, it is clear that clinical diagnosis has substantial limitations, but this study quantifies the discrepancy. Well written, although please note my comment below on authorship.<br><br>Specific comments [document page numbering is not consecutive so using top right page numbering instead]:<br>P8L31-34: Can make more succinct with removing portions of this sentence to "However, studies…among young children are lacking…"<br>P8L55: Change 'conducts' to past tense. If CHAMPS is still ongoing, change to 'is conducting.'<br>P8L5-6: Change 'has been' to 'was.'<br>P9L48: Can you clarify if biosamples are also collected within the 24 hours of time of death? If there is a time range within which these samples are collected, please note this.<br>P9L54: Change syringe to plural.<br>P10L14-15: Combine to 'Nasopharyngeal, oropharyngeal, and rectal swabs are sent for…'<br>P10L16: Can you clarify if all involved anatomical pathologists received specific standardized training relevant to this surveillance?<br>P10L22: 'Near the time of the child's death' is vague – can you provide a more specific time period, or range that can be included here? Can you also provide more details on the 'treating clinicians,' such as what proportion are those with a BSc degree or a MBBS degree? This will be helpful in determining the baseline knowledge and clinical diagnostic skills capacity of the staff.<br>P10L27: Note American spelling throughout ('pediatrician'), but if the journal has a preference would follow that.<br>P10L47-48: The ten most common causes of death as determined by DeCoDe panels are based on what data? Is this based on earlier CHAMPS work? Clarification would be helpful.<br>P13L52-54: Can you provide the sensitivity values here in parentheses?<br>P14L13: I would argue first that the diagnostic errors points to the limitations of clinical diagnosis, and is a gap in care that enhanced

	diagnostic approaches can close.<br>P15L15-17: I also wonder whether the determination of sepsis in this study was based on culture sampling via MITS, and how did you differentiate between transient or asymptomatic bacteremia/positive culture from sepsis and SIRS? This may be something to comment on in the discussion/limitations.<br>Author contributions: I note HL is the first author but CB and CAR also were involved in conceptualizing, designing, interpreting,and writing the first draft of the manuscript. In addition, they also independently reviewed diagnostic pairing. Therefore while I am fully supportive of local investigators receiving due credit and recognition for their involvement and effort in such studies, I wonder if in this instance joint first authorship was considered and would more appropriately adhere to ICMJE authorship criteria. I note that last authors CGW and CAR appear to have contributed equally and from this listing I would also wonder the same of the first three authors. If I am mistaken, please rebut and clarify the contributions of the first listed author to make this clear.

VERSION 1 – AUTHOR RESPONSE

Dear Drs. Rohloff and Raman,

Thank you for the opportunity to provide minor revisions to our article, "Clinicopathological discrepancies in the diagnoses of childhood causes of death in the CHAMPS network: An analysis of antemortem diagnostic inaccuracies" (bmjpo-2024-002654). In our attachment here, we have indicated where the corresponding changes were made in the revised manuscript. We are happy to address further questions that may arise.

Please note that in order to present the complex approach that involved sites in seven countries and to accommodate the requested revisions, which greatly strengthened our manuscript, our main text is >2,500 words.

We thank you very much for considering our revised submission. Please do not hesitate to contact us with any questions.
